# Development of a novel benchmark method to identify and characterize best practices in home care across six European countries: design, baseline, and rationale of the IBenC project

**DOI:** 10.1186/s12913-019-4109-y

**Published:** 2019-05-15

**Authors:** Henriëtte G. van der Roest, Liza van Eenoo, Lisanne I. van Lier, Graziano Onder, Vjenka Garms-Homolová, Johannes H. Smit, Harriet Finne-Soveri, Pálmi V. Jónsson, Stasja Draisma, Anja Declercq, Judith E. Bosmans, Hein P. J. van Hout, IBenC project, Dinnus H. Frijters, Karlijn J. Joling, Roberto Bernabei, Davide L. Vetrano, Angelo Carfí, Jacqueline Schoen, Ho Ming Lau, Ho-Young Wisselink, Jenny Bremner, Michele Calabro, Olivia Dix, Usman Khan, Matteo Vezzosi, Sari Jokinen, Berglind Magnúsdóttir, Inga Valgerður Kristinsdóttir, Joonas Sakki, Anja Noro

**Affiliations:** 1 Department of General Practice and Elderly Care Medicine, Amsterdam Public Health research institute, Amsterdam UMC, VU University medical center, Van der Boechorststraat 7, 1081 BT Amsterdam, The Netherlands; 2 LUCAS Centre for Care Research and Consultancy, KU Leuven, Leuven, Belgium; 3 Department of Geriatrics, Neuroscience and Orthopedics, Agostino Gemelli University Hospital, Università Cattolica del Sacro Cuore, Rome, Italy; 4 Department of Economics and Law, HTW Berlin, University of Applied Sciences, Berlin, Germany; 5 Department of Psychiatry, Amsterdam Public Health research institute, Amsterdam UMC, Vrije Universiteit Amsterdam, The Netherlands & GGZ inGeest Specialized Mental Health Care, Research and Innovation, Amsterdam, The Netherlands; 6 Department of Wellbeing, National Institute for Health and Welfare, Helsinki, Finland; 7 Department of Geriatrics, Landspitali University Hospital, and Faculty of Medicine, University of Iceland, Reykjavík, Iceland; 8 Department of Health Sciences, Faculty of Science, Amsterdam Public Health research institute, Vrije Universiteit Amsterdam, Amsterdam, The Netherlands

**Keywords:** Home care, Health care policy, Quality of care, Costs of care utilization, Elderly, Benchmarking, Cross-country comparison

## Abstract

**Background:**

Europe’s ageing society leads to an increased demand for long-term care, thereby putting a strain on the sustainability of health care systems. The ‘Identifying best practices for care-dependent elderly by Benchmarking Costs and outcomes of Community Care’ (IBenC) project aims to develop a new benchmark methodology based on quality of care and cost of care utilization to identify best practices in home care. The study’s baseline data, methodology, and rationale are reported.

**Methods:**

Home care organizations in Belgium, Finland, Germany, Iceland, Italy, and the Netherlands, home care clients of 65 years and over receiving home care, and professionals working in these organizations were included. Client data were collected according to a prospective longitudinal design with the interRAI Home Care instrument. Assessments were performed at baseline, after six and 12 months by trained (research) nurses. Characteristics of home care organizations and professionals were collected cross-sectionally with online surveys.

**Results:**

Thirty-eight home care organizations, 2884 home care clients, and 1067 professionals were enrolled. Home care clients were mainly female (66.9%), on average 82.9 years (± 7.3). Extensive support in activities of daily living was needed for 41.6% of the sample, and 17.6% suffered cognitive decline. Care professionals were mainly female (93.4%), and over 45 years (52.8%). Considerable country differences were found.

**Conclusion:**

A unique, international, comprehensive database is established, containing in-depth information on home care organizations, their clients and staff members. The variety of data enables the development of a novel cost-quality benchmark method, based on interRAI-HC data. This benchmark can be used to explore relevant links between organizational efficiency and organizational and staff characteristics.

**Electronic supplementary material:**

The online version of this article (10.1186/s12913-019-4109-y) contains supplementary material, which is available to authorized users.

## Background

Health care systems in Europe are evolving. Health care for dependent older people is especially subject for major changes. Due to the rapidly greying population and the projected shrinking work force, the old-age dependency ratio in Europe is expected to almost double from 29.6% in 2016 up to 52.3% in 2080 [1]. In combination with an increased demand for long-term care in older people, this development will put a strain on the sustainability of healthcare systems. Care quality might not be guaranteed in the future. In addition future cost of elderly care also are a concern [2, 3]. In order to save costs, European health care policy shifted from institutionalization to ageing in place. Although costs of home care are generally lower than those of institutionalized care, the long-term impact of such a shift in care delivery between settings is unclear with regard to potential changes in case-mix of community and institutional care settings, quality, and eventually on public costs of long-term care for older people.

Demographic ageing varies within Europe, the share of people aged 65 years or older ranges from 22.0% in Italy to 13.2% in Ireland [1], and large international variation in dependency levels in long-term care settings exists [4–6]. As a response to address the local health care needs of elderly populations, different national policy decisions, legislations, and reimbursement systems emerged, which led to a broad range in arrangements of pan-European care delivery [7–9] and differences in care performance [4, 8, 10]. The multifaceted diversity of home care delivery in Europe is a source of valuable information to serve the imminent reform of home care systems.

In his integrated model on quality, costs, and health, Donabedian [11] states that care costs and quality vary according to the strategy used by the care provider, but also that improvements in the efficiency of organization and delivery of care can lead to lower costs. In other words, the structure of, and applied processes within care organizations can foster or confine high quality care delivery [12]. However, studies on this topic are scarce, dispersed across health care settings, and the results are inconsistent. Findings showed that amongst others caseload size [13], staffing rations [14–16], turnover rate, and staff mix [16], were found to be related to quality, and sometimes inversely to costs of care [17]. In addition characteristics and perceptions of care professionals, such as job satisfaction, were found to be associated with quality of care [18, 19]. In order to reliably compare outcomes of community care, such a benchmark needs to rely on high quality data which generate valid indicators for quality and generate realistic estimates of costs of care utilization. Up to now, a comprehensive data base on quality and costs of care utilization of home care, including demographic, functional, psychological, and social information on home care clients, structure and process information on home care organizations, and information on staff does not exist.

The ‘Identifying best practices for care-dependent elderly by Benchmarking Costs and outcomes of Community Care’ (IBenC) project aims to bridge this gap. Based on comprehensive data, a methodology will be developed to identify best practices in home care delivery for care dependent community dwelling elderly people by benchmarking costs and performance of home care delivery systems across Europe, taking into account micro (client), meso (organizational) and macro (policy) levels in home care [20]. The results can provide valuable insights for health care policy makers in order to support decision making towards sustainable health care for older people.

The primary aim of this paper is to provide the methodology of the IBenC study, to describe the baseline characteristics of included home care organizations, home care clients, and home care staff, and to outline the rationale and further steps in the development of the benchmark method.

## Methods

### Design

Data collection was performed between January 2014 and August 2016 in selected regions of six European countries: Belgium, Finland, Germany, Iceland, Italy, and the Netherlands. Data were collected in parallel amongst three target groups: home care organizations, home care clients, and home care professionals. Characteristics of care organizations and home care staff were collected according to a cross-sectional design. Data collection amongst home care clients followed a prospective longitudinal design with assessments at baseline, six, and 12 months. Ethical approval for the study was obtained from authorized medical ethical committees according to local regulations in each of the participating countries. Belgium (Flanders): Commissie Medische Ethiek van de Universitair Medische Ziekenhuizen Katholieke Universiteit Leuven, No. ML10265; Finland: Tutkimuseettinen työryhmä, No. THL/796/6.02.01/ 533/2014; Germany: Ethikkommission des Institut für Psychologie und Arbeitswissenschaft der Technische Universtität Berlin, No. GH_01_20131022; Iceland: Vísindasiðanefnd, No. 13–176-S1; Italy: Comitato Etico Università Cattolica del Sacro Cuore, No. 2365/14; The Netherlands: Medical Ethics Review Committee VU University Medical Center, No. 2013.333.

Written consent was sought from participants to the study according to local regulations. Only for clients from home care organizations that used interRAI-HC in routine care, informed consent was not required. These assessments were performed for clinical purposes by organizations’ own staff, and data were transferred anonymously to the national study centers. In none of the participating countries jurisdiction required informed consent for the use of anonymous clinical data for secondary research purposes. Informed consent from professional caregivers was obtained digitally before starting the online questionnaire.

### Setting and sample

Home care organizations in selected areas that delivered health and/or social care in the community were invited to participate. Data heterogeneity was required for development of the benchmark method, therefore organizations were selected on variety in care practice, rather than on representativeness. Care organizations preferably used the interRAI-HC [21–23] in routine care practice.

All members of the professional care staff working at the enrolled organizations were eligible for participation in the study.

Eligibility criteria for home care clients were: receive home care from enrolled organizations, 65 years or older, and expected to remain in care for at least six months after inclusion. Exclusion criteria were: terminal illness, short term care, planned institutionalization within six months, cognitive impairment (score Cognitive Performance Scale (CPS) ≥ 3) without known informal caregiver or legal representative. Calculation of the sample size was based on the least prevalent quality indicator (QI) ‘neglect or abuse’ in the Aged in Home Care (AdHOC) project [4]. Respondents in the AdHOC study were very similar to the targeted population of the IBenC study, since AdHOC was conducted among people of 65 years and older receiving home care in11 European countries. The prevalence of the QI ‘neglect and abuse’ in the AdHOC population was 4.4%, while anticipating a dropout of 20% and a refusal rate of 7% until the end of the study [10], a preferable minimum sample size of 153 clients per organization, and three organizations per country was calculated for IBenC. A total cohort of 2750 home care clients was envisaged, in which 459 home care clients per country were included.

### Outcome measures

#### Characteristics of home care organizations

A questionnaire on structures and processes of home care organizations was developed based on evidence based literature about care organizations and quality of care (see Additional file 1). Face validity of the English version of the questionnaire was tested by three home care managers and seven academic experts from the six participating countries. Questions were reviewed for applicability, clarity, and completeness. The final questionnaire was translated into the different languages required for the project, and subsequently back-translated into English. The main themes addressed are structural features (e.g. type and ownership, service region size), caseload (e.g. number of home care clients aged 65 years and over), staff characteristics (e.g. discipline mix), and processes (e.g. meetings and accountability) of home care organizations. More details on the questionnaire are described elsewhere [24].

#### Characteristics of home care clients

Background characteristics of home care clients were assessed at baseline with the comprehensive geriatric assessment instrument interRAI-HC [21, 23]. This instrument is part of a suite of instruments that are globally used in routine care to support assessment and care planning in health care settings for vulnerable patient groups. The interRAI-HC allows for continuous assessment of medical, psychological, social, and functional capabilities and needs of care dependent older people living in the community [21–23]. Validated language versions of the interRAI-HC were available in all languages required [25]. To measure impairment levels, interRAI-HC multi-item scales were used. Functional status was evaluated with the Activities of Daily Living Hierarchy scale (ADLH), ranging from 0 (no impairment) to 6 (total dependence) [26]. A score of ≥3 indicates that extensive ADL support is required. The Instrumental Activities of Daily Living Performance scale (IADLP) was used to assess self-performance of eight IADL tasks that could be rated from independent (0) to total dependence (6). The IADLP scale cumulates these scores and ranges from 0 (independent) to 48 (total dependence) [22]. Cognitive status was assessed with the 7-point Cognitive Performance Scale (CPS) [27]. Cut off scores of ≥3 were used to indicate the presence of moderate to severe impairment. The presence of depressive symptoms was assessed by means of the 7-point Depression Rating Scale (DRS) [28], a score of ≥3 indicates the presence of minor or major depressive disorders. To assess pain the 4-point Pain scale was used. A score of ≥2 indicated daily pain in a home care client [29]. Higher scores on all scales indicate higher levels of impairment.

#### Characteristics of professional caregivers

A questionnaire to assess staff characteristics in home care was developed based on evidence-based literature on job satisfaction in home care settings for older people [24] (see Additional file 2). In addition to demographic, social, and job characteristics, the questionnaire mainly consisted of pre-existing validated questionnaires and scales on psychosocial work environment factors: e.g. degrees of freedom, job insecurity, salary, social relationships at work, job satisfaction [30]; burn-out [31]; intention to turn-over [32]; dissatisfaction with work-schedule [33]; physical workload [30]; and type of care delivery [34, 35]. The same procedure used for the questionnaire on home care characteristics was applied to test for face validity of this questionnaire and to conduct the translations.

### Procedure

#### Enrolment procedure

Based on convenience and diversity between home care organizations, local study centers selected and invited home care organizations to participate in the study. Standardized written information about the study was adapted for country specific situations. Individual site visits were made by the principal investigators to provide project specific information to key persons within care organizations.

Eligible home care clients were selected from the enrolled organizations’ caseloads. Home care clients were informed in writing and orally about the study and invited to participate. Home care clients signed an informed consent form before entering the study. According to local regulations, informed consent was not required for participants from organizations using the interRAI-HC in routine care practice.

All staff members of the enrolled care organizations in Belgium, Finland, Iceland, Italy, and the Netherlands, and professionals of four German home care organizations, received information and an invitation for the study via the conventional communication channel within their organization (e-mail or postal mail). For this process the Total Design Method approach was applied, non-responders received up to three reminders [36]. Invitations contained an individual login code and password for the online questionnaire. Participation was voluntary. Informed consent was obtained from every respondent digitally or in writing before starting the questionnaire.

#### Data collection

Organizational characteristics were collected in an online questionnaire via key persons (e.g. manager, director) of participating care organizations. Administration of the organization questionnaire took approximately 45 to 60 min. If required, assessment support from the local study center was provided.

Patient outcome data were collected at the dwellings of home care clients by trained (research) nurses, using licensed software [25]. All assessors followed a standardized training on the interRAI-HC (information available with the first author). In all countries but Italy, data collection took place prospectively. Assessment errors were detected in the Italian data, which made the longitudinal data unfit for use. Overall data collection was in its’ final phase and time constraints prohibited collection of new longitudinal routine care data in Italy. Therefore was decided to include recent client assessments serving as six month follow-up data, and retrospectively select baseline assessments. For completion of the interRAI-HC all sources of information are used: patient interviews, care files, observation, and information obtained from informal and formal caregivers. Duration of an interRAI-HC assessment is on average 60 min.

Care staff characteristics were collected with an online self-report questionnaire. Upon request the questionnaires could also be filled out on paper. Administration of the staff characteristics questionnaire took approximately 30 min.

### Analytical approach

Analyses were performed with SPSS version 22.0. Descriptive statistics and frequencies were performed to report on the baseline sample. Country differences for continuous variables were analyzed using Analysis of Variance tests (ANOVAs) and Kruskal-Wallis tests in case of unequal variance. For categorical variables Chi square tests were performed. IBenC data released in December 2016 were used for the study.

## Results

### Response and retention

Overall response was high: 38 (92.7%) home care organizations, 2884 (86.5%) home care clients, and 1067 (60.1%) professional staff members consented to participate. During the study 666 home care clients were discharged (23.1%), of whom 65 did not need care anymore (2.2%), 233 were admitted to a long-term care facility (8.1%), 48 to acute care (1.7), and 200 home care clients died (6.9%). In total 737 home care clients were lost for follow-up (25.5%). The percentage of home care clients lost for follow-up were especially high in the Netherlands (46.0%) and in Italy (91.2%). Software problems caused one Dutch organization to temporarily stop interRAI-HC assessments between baseline and six months, and at 12 months assessments started up again. Therefore, few clients of this home care organization that were included at baseline were assessed at follow-up. Due to the retrospective selection process in Italy, all included home care clients had received care for at least six months. Between baseline and six month follow-up no discharge events, such as admission to a long-term care institution or death, took place. Because of time constraints in data collection, only 44 of all included home care clients were assessed at 12 month follow-up. The Italian home care clients that could not be assessed at 12 month follow-up, were considered lost for follow-up. The percentage lost for follow-up in the other countries ranged from 0.5 to 9.5%.

### Home care organizations

In total 38 home care organizations participated in the study: 18 in Belgium, three in Finland, 11 in Germany, one in Iceland, two in Italy, and three in the Netherlands. Characteristics of 36 home care organizations are presented in Table 1. The majority of the organizations were privately owned (83.3%) of which 16.7% were for profit organizations. Home care organizations provided predominantly nursing care (55.6%). A proportion of 16.7% of the organizations operated exclusively in rural areas. Organization size varied considerably, the average number of licensed practical and (advanced practice) registered nurses practicing within an organization ranged between 33.9 up to 4224.0 per country.

**Table 1:** Characteristics home care organizations

	Total	Belgium	Finland	Germany	Iceland	Italy	The Netherlands	*p-value*
	*n* = 36	*n* = 18	n = 3	*n* = 9	n = 1	*n* = 2	*n* = 3	
	(%)	(%)	(%)	(%)	(%)	(%)	(%)	
Public ownership	6 (16.7)	0 (0.0)	3 (100.0)	0 (0.0)	1 (100.0)	2 (100.0)	0 (0.0)	0.000
Private ownership	30 (83.3)	18 (100.0)	0 (0.0)	9 (100.0)	0(0.0)	0 (0.0)	3 (100.0)	
Not for profit	25 (83.3)	18 (100.0)	0 (0.0)	5 (55.6)	0 (0.0)	0 (0.0)	2 (66.7)	0.010
Mainly nursing care	20 (55.6)	18 (100.0)	0 (0.0)	0 (0.0)	0 (0.0)	0 (0.0)	2 (66.7)	0.000
Mix nursing and social care	16 (44.4)	0 (0.0)	3 (100.0)	9 (100.0)	1 (100.0)	2 (100.0)	1 (33.3)	
Years since foundation (mean ± SD)	32.8 ± 19.3	47.2 ± 14.6	6.0 ± 4.6	23.6 ± 7.3	6.0	33.0 ± 5.7	10.3 ± 4.2	0.000
Rural area	6 (16.7)	3 (16.7)	0 (0.0)	2 (22.2)	0 (0.0)	0 (0.0)	1 (33.3)	0.060
Number of clients								
≤ 100	10 (24.9)	6 (33.3)	0 (0.0)	4 (57.1)	0 (0.0)	0 (0.0)	0 (0.0)	0.003
101–500	6 (17.6)	3 (16.7)	0 (0.0)	3 (42.9)	0 (0.0)	0 (0.0)	0 (0.0)	
501–1000	10 (29.4)	8 (44.4)	2 (66.7)	0 (0.0)	0 (0.0)	0 (0.0)	0 (0.0)	
> 1000	8 (23.5)	1 (5.6)	1 (33.3)	0 (0.0)	1 (100.0)	2 (100.0)	3 (100.0)	
Average team size (mean ± SD)	5.5 ± 1.7	4.9 ± 1.0	7.0	4.9 ± 1.0	7.0 ± 0.0	8.5 ± 0.7	6.7 ± 4.0	0.000
Number of nurses (mean ± SD)	399.0 ± 1519.5	33.9 ± 23.6	127.3 ± 85.2	N/D	135.0	N/D	4224.0 ± 4596.2	0.000
Organisation provides case management	22 (62.9)	13 (76.5)	3 (100.0)	1 (11.1)	1 (100.0)	2 (100.0)	2 (66.7)	0.009
Organisation checks eligibility criteria client after referral	11 (30.6)	4 (22.2)	3 (100.0)	0 (0.0)	1 (100.0)	2 (100.0)	1 (33.3)	0.003
Always care professional on call	24 (66.7)	16 (88.9)	2 (66.7)	2 (22.2)	1 (100.0)	1 (50.0)	2 (66.7)	0.026
Organisation assesses quality of care	29 (80.6)	11 (61.1)	3 (100.0)	9 (100.0)	1 (100.0)	2 (100.0)	3 (100.0)	0.122
Voluntary	7 (25.0)	4 (40.0)	1 (33.3)	0 (0.0)	0 (0.0)	1 (50.0)	1 (33.3)	0.073
Legal obligation	10 (35.7)	1 (10.0)	0 (0.0)	7 (77.8)	1 (100.0)	1 (50.0)	0 (0.0)	
Both voluntary and legal obligation	11 (39.3)	5 (50.0)	2 (66.7)	2 (22.2)	0 (0.0)	0 (0.0)	2 (66.7)	

*N/D* No data

### Home care clients

A total of 2884 home care clients were enrolled in the study at baseline, with relatively equal sample sizes per country: 525 (18.2%) from Belgium, 456 (15.8%) from Finland, 493 (17.1%) from Germany, 420 (14.6%) from Iceland, 499 (17.3%) from Italy, and 491 (17.0%) from the Netherlands. Two third of the home care clients were female, the average age was 82.9 (± 7.3). Almost one third of the participants was married or with partner (31.0%), the majority of the participants lived alone (57.7%). Significant country differences were found (*p* < 0.05). On average 17.6% of the respondents suffered from moderate to severe cognitive decline, ranging from 1.8% in the Netherlands up to 37.1% in Italy. Extensive ADL support was required for 41.4% of the respondents, the proportion of respondents in need for this was low in Iceland (8.3%), but very high in Italy and Belgium (respectively 80.3 and 82.0%). The average amount of professional home care, the total amount of received health aid, nursing, and domestic care, was 5.1 (± 6.1) hours per week, and was the lowest in Italy (1.0 ± 2.6). Italian home care clients received the highest amount of informal care, 23.2 (± 17.2) hours in the last three days, whereas this was the lowest in Finland (5.9 ± 13.5). See Table 2.

**Table 2:** Baseline characteristics of home care clients

	Total	Belgium	Finland	Germany	Iceland	Italy	The Netherlands	*p-value*
	*n* = 2884	*n* = 525	*n* = 456	*n* = 493	*n* = 420	*n* = 499	*n* = 491	
	(%)	(%)	(%)	(%)	(%)	(%)	(%)	
Age, years (mean ± SD)	82.9 ± 7.3	82.4 ± 6.7	82.7 ± 7.0	84.2 ± 7.6	83.7 ± 7.0	81.8 ± 7.9	82.5 ± 7.1	0.000
Female	1930 (66.9)	352 (67.4)	313 (68.6)	351 (71.2)	292 (69.5)	286 (57.3)	336 (71.0)	0.000
Married/partner	815 (31.0)	179 (34.9)	71 (15.5)	124 (25.3)	129 (30.7)	203 (45.0)	109 (35.9)	0.000
Living alone	1656 (57.7)	252 (48.9)	369 (80.9)	359 (72.8)	256 (61.0)	82 (16.4)	338 (69.4)	0.000
At least one informal caregiver present	2455 (85.1)	482 (100.0)	381 (83.6)	292 (59.2)	417 (99.3)	496 (99.4)	387 (78.8)	0.000
Informal care, hours last three days (mean ± SD)	11.6 ± 16.4	N/D	5.9 ± 13.5	8.3 ± 14.0	8.8 ± 14.7	23.2 ± 17.2	7.9 ± 14.2	0.000
Professional care, hours last seven days (mean ± SD)	5.1 ± 6.1	8.5 ± 7.8	5.1 ± 5.2	7.5 ± 6.9	3.6 ± 3.8	1.0 ± 2.6	4.6 ± 4.7	0.000
Extensive ADL support (ALDH ≥3)	1181 (41.4)	419 (82.0)	60 (13.2)	238 (48.4)	35 (8.3)	388 (80.3)	41 (8.4)	0.000
Moderate to severe cognitive impairment (CPS ≥ 3)	503 (17.6)	91 (17.9)	49 (10.7)	135 (27.4)	40 (9.5)	179 (37.1)	9 (1.8)	0.000
Depressive symptoms (DRS ≥ 3)	595 (20.9)	131 (25.6)	55 (12.1)	114 (23.2)	73 (17.4)	106 (21.9)	116 (23.6)	0.000
Daily pain (Pain scale ≥ 2)	725 (25.3)	99 (19.6)	124 (27.3)	104 (21.1)	139 (33.1)	105 (21.0)	154 (31.4)	0.000
IADL performance scale (0–48) (mean ± SD)	28.9 ± 14.2	34.0 ± 10.7	26.4 ± 13.0	28.7 ± 14.9	23.8 ± 11.5	39.3 ± 11.7	17.0 ± 12.3	0.000
IADL performance								
Maximum assistance or total dependence in ≥ 4 out of 8 tasks	1085 (42.0)	301 (62.1)	173 (43.4)	191 (42.7)	154 (36.8)	188 (38.9)	78 (22.4)	0.000
Maximum assistance or total dependence in all 8 tasks	390 (15.1)	71 (14.6)	19 (4.8)	69 (15.4)	10 (2.4)	215 (44.5)	6 (1.7)	

*N/D* No data

### Care professionals

In total 1067 care professionals from 31 organizations participated. Demographics of the professional caregivers are displayed in Table 3. The majority of the respondents was female (93.4%) and older than 45 years (52.8%). Half of the respondents worked full time (52.8%), with the lowest proportion in the Netherlands (8.2%) and the highest proportions in Italy and Finland (respectively 97.7 and 95.6%). In Germany, the respondents experienced the highest level of work related burnout, the highest physical workload and work pace and were least satisfied in their job as compared to the other countries. The Dutch respondents in comparison to care professionals from other countries, experienced the lowest level of work related burnout, the lowest work pace, and were most satisfied with their job and payment. Significant country differences were found for all variables reported in Table 3 (*p* < 0.05).

**Table 3:** Home care staff characteristics

	Total	Belgium	Finland	Germany	Iceland	Italy	The Netherlands	*p-value*
	*n* = 1067	*n* = 401	*n* = 272	*n* = 80	*n* = 105	*n* = 43	*n* = 166	
	(%)	(%)	(%)	(%)	(%)	(%)	(%)	
Female	984 (93.4)	384 (96.0)	264 (97.8)	63 (778.8)	99 (97.1)	29 (67.4)	145 (91.8)	0.000
Age < 30 years	182 (17.3)	91 (22.8)	48 (17.7)	10 (13.0)	16 (15.7)	5 (11.6)	12 (7.6)	0.000
30–45 years	314 (29.9)	135 (33.8)	75 (27.7)	26 (33.8)	21 (20.6)	17 (39.5)	40 (25.3)	
> 45 years	555 (52.8)	174 (43.5)	148 (54.6)	41 (53.2)	65 (63.7)	21 (48.8)	106 (67.1)	
Position								
Registred/Advanced practice registred nurse	713 (67.5)	298 (74.5)	191 (70.2)	62 (77.5)	22 (21.6)	13 (30.2)	127 (79.9)	0.000
Licenced practical nurse	184 (17.4)	38 (9.5)	67 (24.6)	0 (0.0)	48 (47.1)	17 (39.5)	14 (8.8)	
Nursing assistant	26 (2.5)	0 (0.0)	0 (0.0)	9 (11.3)	16 (15.7)	0 (0.0)	1 (0.6)	
Manager	57 (5.4)	35 (8.8)	10 (3.7)	5 (6.3)	2 (2.0)	5 (11.6)	0 (0.0)	
Degree								
Secondary school to BSc	369 (35.4)	175 (44.1)	72 (26.7)	9 (11.3)	43 (45.7)	22 (51.2)	48 (30.2)	0.000
BSc and higher	332 (31.9)	177 (44.6)	29 (10.8)	3 (3.8)	25 (26.6)	16 (37.2)	82 (51.6)	
Experience home care, years (mean ± SD)	11.6 ± 10.0	14.2 ± 11.2	10.8 ± 9.8	8.2 ± 7.2	8.8 ± 6.6	10.2 ± 7.4	10.4 ± 8.8	0.000
Permanent contract	906 (87.5)	375 (95.4)	189 (71.6)	65 (83.3)	88 (87.1)	40 (95.2)	149 (94.3)	0.000
Full time work arrangement	558 (52.8)	125 (31.3)	260 (95.6)	67 (83.8)	51 (50.0)	42 (97.7)	13 (8.2)	0.000
Sick leave								
None past year	341 (32.6)	152 (38.6)	66 (24.5)	35 (43.8)	26 (26.0)	7 (16.3)	55 (34.6)	0.000
> 1 month past year	143 (13.7)	53 (13.4)	36 (13.4)	24 (30.0)	11 (11.0)	2 (4.7)	19 (11.9)	
Perception of work related burn-out^a^ (mean ± SD)	33.6 ± 16.7	30.9 ± 14.8	38.8 ± 16.8	43.0 ± 16.9	32.3 ± 14.7	42.3 ± 17.7	25.2 ± 16.4	0.000
Perception of physical workload^b^ (mean ± SD)	5.8 ± 2.6	3.1 ± 2.6	5.9 ± 2.2	6.8 ± 3.3	6.0 ± 2.5	4.1 ± 2.7	4.4 ± 1.9	0.000
Perception of work pace^a^ (mean ± SD)	65.3 ± 19.2	64.8 ± 17.6	70.2 ± 16.7	77.4 ± 20.5	64.3 ± 17.5	74.7 ± 12.3	50.0 ± 19.8	0.000
Perception of payment^a^ (mean ± SD)	40.8 ± 27.2	49.0 ± 22.6	26.8 ± 24.2	38.4 ± 29.8	26.6 ± 26.2	34.9 ± 28.4	56.4 ± 26.1	0.000
Perception of job satisfaction^a^ (mean ± SD)	71.5 ± 18.7	73.9 ± 15.5	64.8 ± 21.9	63.7 ± 11.0	70.5 ± 17.4	72.1 ± 16.2	81.1 ± 19.0	0.000

^a^Scale range 0–100 (low to high)

^b^Scale range 0–12 (low to high)

## Discussion

The IBenC project established a unique and comprehensive database on home care. It comprises international longitudinal data on functioning of home care clients, from which care performance, and cost of care utilization information can be generated. In addition, information on organizational structure and processes, and characteristics of care professionals can be linked to client data. The data clearly show existing differences in populations and care arrangements between countries and organizations included in the study. This variety is a prerequisite for development and piloting of the benchmark.

Although the findings give an indication of the overall state of home care in the participating countries, they need to be interpreted with caution. Only a limited number of home care organizations per country was included, and selection was based on variety in size, care practice, and location. Consequently, they may not be representative for all countries involved. The differences found between countries to a certain extent can be attributed to macro and local policies. In Belgium, the responsibility for home care is largely located at a federal and community level, and in Germany this is mainly at a federal and municipal level. In both countries many private for-profit and not-for-profit home care organizations operate in a competing market [37, 38]. This leads to smaller organizations than for instance in Iceland, where home care organizations are owned by the government. Accessibility to home care varies substantially across Europe, and depends on factors such as availability of care providers, reimbursement systems, and expectations of informal care involvement. The utilization rate of home care, serves as an indicator for accessibility. In the countries involved in the study, the highest proportion of home care recipients is seen in the Netherlands, followed by Belgium, Finland, Italy and Germany [38]. High accessibility may result in a relatively low dependency level in home care clients. This is seen in the Dutch sample, where accessibility to home care is considered to be high. In contrast, accessibility to home care in Germany and Italy is considered to be much lower. The German long-term care system does not cover all care. Entitlement to care is needs tested, and persons whose level of dependency falls below a needs threshold do not receive benefits, or insufficient resources to meet their needs. For a substantial amount of persons in need of home care, access to care depends on their social support and private resources. In Italy, access to home care is not only needs-tested, but also depends on means and availability of informal care [38, 39]. The levels of cognitive and functional impairment of home care clients from these countries was on average higher than those found in the Netherlands.

Since accurate data on European home care populations is unavailable, it is uncertain to what extent the profiles of the home care clients are representative. However, impairment levels of the IBenC sample can be related to results of the AdHOC study. This study was performed amongst home care clients in all IBenC countries, except Belgium, and sought for representativeness of nation’s urban areas in their sampling [4]. The average levels of cognitive and functional impairment presented in home care clients in IBenC are in line with AdHOC findings. In Italy, home care clients displayed the highest care needs, while home care clients in Finland and the Netherlands showed the lowest levels of cognitive and functional decline. Also the amount of utilized professional care within the IBenC sample is fairly in line with AdHOC results. In Italy formal care utilization is low. Due to large moral and legal responsibilities families have towards care for its elderly members, care is largely provided by informal caregivers [37]. This responsibility is lower in Northern European countries, consequently the amount of formal care utilization in these countries is higher. It must be noted that overall impairment levels found in IBenC were higher than in AdHOC. AdHOC’s data collection took place over a decade before IBenC started. These differences might be the result of changes in European health care policy in supporting people to age in place. People live independently for a longer period of time, but with higher dependency levels.

Similar regional dependency patterns were also found in the longitudinal Survey of Health, Ageing and Retirement in Europe (SHARE) project, involving 17 European countries [40]. A cross-sectional comparison of dependency in a large cohort of people aged 65 to 84 was performed over the year 2015. Highest disability levels were found in the Southern European regions, followed by the Central and Northern regions [40]. However, a specific comparison amongst home care recipients was not made.

A selection bias may have occurred in the Italian and Dutch samples. Since data in Italy were collected retrospectively, the sample consisted mainly of home care ents receiving long-term care. Probably home care clients relying chronically on home care services may be overrepresented in the sample, and disability levels could be higher than expected. In the Netherlands, the level of cognitive impairment was very low. This probably derived from the recruitment process in two of the three sites. One of the main reasons provided for refusal was cognitive impairment. Home care clients with more advanced cognitive decline are likely to be underrepresented in the Dutch sample.

Although almost all home care organizations recruited participants amongst their staff, we remain uncertain about the representativeness of the professional caregivers. In Germany, many home care organizations refused to participate, since the questionnaire for staff members interfered with the mandatory commitment to conduct regular staff satisfaction surveys. Also nursing assistants may be underrepresented in the samples. The willingness to participate was especially low amongst them. In addition, the Belgian, Italian and two of the Dutch home care organizations did not employ nursing assistants.

### Benchmark development

The richness of, and the variety in the IBenC database will add to the current knowledge on the relationship between home care organization characteristics, performance, and costs of care. By using a single instrument approach, a new benchmarking method on both quality and costs of care utilization will be developed. To determine the two pillars of the benchmark, quality of care and cost of care utilization, longitudinal data are needed. For this purpose baseline and six month follow-up interRAI-HC assessments shall be used.

Quality of care will be expressed by quality indicators (QIs). At an aggregated level, the interRAI-HC generates 23 case-mix and risk adjusted home care QIs that reflect organizational performance with respect to functioning, clinical status, social life, distress, and service use [41–43]. In order to make meaningful comparisons of organizational performance, stratification and adjustment for differences in client profiles will be carried out as an important component of calculating the QIs [43, 44].

Quality of care is a multidimensional concept. In home care, often complex and diverse needs have to be met in order to provide good care. This is reflected by the large number of QIs, which are extremely helpful for quality improvement trajectories [45, 46]. For a concise overview of organizational performance, two 11-point summary scales will be used in the benchmark [43]. The Home Care Clinical Balance Quality scale (HC-CBQS) is generated from nine individual QIs and reflects performance on return to clinical balance of clients, i.e. improvement of functioning and psychosocial wellbeing. The second scale, the Home Care Independence Quality scale (HC-IQS), reflects organizational performance in the domains of functional independence and engagement and is generated by 11 QIs [43]. Both scales have high internal consistency and distinguish for care performance at a macro level [43, 44].

Cost of care will be calculated adopting a societal perspective, i.e. taking into account informal care costs in addition to health care costs. The interRAI-HC assesses care utilization from a client perspective, registering systematically the amount of home care, physician visits, other health care services (e.g. physical therapy), hospital admissions and emergency room visits, supportive care services, institutional care, and informal care. The amount of consumed care will be valued according to standard prices, enabling viable comparisons between organizations in different countries. The interRAI-HC is found to have good convergent validity for cost of care assessments as compared to the Resource Utilization in Dementia - Lite Version instrument (RUD-Lite) [47, 48]. Adjustments for case-mix differences will be applied in order to generate comparable costs of care utilization. For the purpose of the benchmark, mean case-mix adjusted societal care costs will be estimated for home care organizations.

Since the HC-CBQS and HC-IQS reflect quality of different types of care, as demonstrated in Morris et al. [43] and Foebel et al. [44], organizational performance on both scales will be integrated with their respective cost of care utilization estimates, creating two benchmarks. These integrated measures will reflect organizational efficiency, e.g. how well home care organizations perform on improvement (HC-CBQS), and on the other hand on maintenance of functioning, and prevention of decline of their clients (HC-IQS), while taking into account the care costs they generate. By setting references within the benchmark for acceptable quality of care and costs of care utilization, organizational efficiency can be determined. Organizations with higher scores on the summary quality scales than what is set for acceptable quality, and generating on average lower costs of care utilization than the reference costs, can be considered efficient. Less efficiency is demonstrated when care performance is better than the reference, but average costs are higher than the acceptable costs, or when costs of care utilization are low, but quality of delivered care is inferior too. The highest level of inefficiency is manifested when costs are higher than the reference costs, and organizational performance is lower than what is considered acceptable quality of care.

Once the efficiency of the organizations is determined, the relationship between the level of efficiency, with organizational features, and characteristics of professional caregivers will be explored. Associations between organizational efficiency and potentially modifiable organizational or staff characteristics will provide pointers for improvement.

Although the number of home care organizations within the IBenC project is too small to generate robust evidence on which factors contribute to efficiency, this benchmark methodology has good potential for swift expansion and implementation. Since interRAI-HC assessments are globally used in routine care practice, data from which quality and cost of care information can be derived are already being administered in many home care organizations. A large benchmark will generate powerful information to support health care policy makers and care organizations in reforming home care according to best practices. Better insight into the functioning of home care organizations increases the odds that our health care system becomes sustainable for the future, without having to compromise on quality.

### Strengths and limitations

The systematic method used in IBenC to build a database on home care, distinguishes itself from other large (inter)national databases as the Organisation for Economic Co-operation and Development (OECD) [49] or the Canadian Institute for Health Information (CIHI) [50]. These databases merely rely on aggregated data from different sources. Cost of care expenses are often derived from public sources, while quality of care information is derived from client information, or by so called process indicators. These databases, similar as with the IBenC database, generate relevant health care information for policy makers on macro levels. However, the systematic manner to collect information on the macro, meso, and micro level of health care systems, as applied in IBenC and the possibility to reliably link this leveled information, provides more rich and powerful information for a thorough insight into the functioning of home care systems.

Another major advantage of the applied method is that data collected over the same time period with a single, validated instrument, generate both cost and quality information. The interRAI-HC enables case-mix corrections, therefore ensuring comparability. Especially in international economic evaluations, data are not comparable due to reasons as variation in inclusion and categorization of costs categories [51, 52]. In most home care organizations interRAI-HC data were collected in routine care. Since this information is used in the primary care process and provides important input for care plans, the likelihood of capturing the actual and complete situation on care performance within organizations with this data is very high. The lack of standardized instruments to capture characteristics of home care organizations, and the limited knowledge on organizational determinants for quality and costs of care in home care settings on the other hand, form a limitation for the study. It was necessary to use a broad scope in both the questionnaires for home care organizations and staff, in order to capture as many potentially relevant aspects as possible. During the data collection process it became clear that some organizations found difficulties answering certain items, since the information was not available or could not be generated from administrative systems. Many organizations experienced difficulties to provide for example reliable data on the number and full time equivalents (FTE) of permanent and temporary staff, or educational levels of staff by FTEs. Since quality of care provisioning varies between home care organizations [13], more effort is needed in the development of validated questionnaires for home care, that capture organizational characteristics relevant for both quality and costs of delivered care in a feasible manner.

## Conclusion

Insight into the relationships between organizational efficiency and organizational and staff characteristics is essential in a care environment of increasingly complex client populations, growing caseloads, and restricted budgets. The study showed large variety between countries in samples of home care clients, home care organizations and staff. By developing a benchmark method based on sound data on costs and quality of care, the IBenC project hopes to contribute to existing knowledge of the functioning of home care organizations.

## Additional files

Additional file 1: **Appendix 1.** IBenC project: Questionnaire 2: characteristics of the home care organisations. Questionnaire developed within the IBenC project to assess structural and process characteristics of home care organizations. (DOCX 92 kb)

Additional file 2: **Appendix 2.** IBenC project: Questionnaire 1- characteristics of the care giving staff. Questionnaire developed within the IBenC project to assess characteristics of professional caregivers working in the participating home care organizations. (DOCX 72 kb)

## Abbreviations

AdHOC: AgeD in the Home Care
ADLH: Activities of Daily Living Hierarchy scale
ANOVAs: Analysis of Variance tests
CIHI: Canadian Institute for Health Information
CPS: Cognitive Performance Scale
DRS: Depression Rating Scale
FTE: Full time equivalent
HC-CBQS: Home Care Clinical Balance Quality scale
HC-IQS: Home Care Independence Quality scale
IADLP: Instrumental Activities of Daily Living Performance scale
IBenC: Identifying best practices for care-dependent elderly by Benchmarking Costs and outcomes of Community Care
interRAI-HC: interRAI Home Care instrument
interRAI-LTCF: interRAI instrument for Long Term Care Facilities
MDS-HC: Minimum Data Set for Home Care
OECD: Organisation for Economic Co-operation and Development
QI: Quality indicator
RUD-Lite: Resource Utilization in Dementia - Lite Version
