# Supplementary material for: Development of a novel benchmark method to identify and characterize best practices in home care across six European countries: design, baseline, and rationale of the IBenC project
Source: BMC Health Serv Res. 2019 May 15;19:310. doi: 10.1186/s12913-019-4109-y (PMC6521361; doi:10.1186/s12913-019-4109-y)
Supplement: Supplementary file 1 — Appendix 1. IBenC project: Questionnaire 2: characteristics of the home care organisations. Questionnaire developed within the IBenC project to assess structural and process characteristics of home care organizations. (DOCX 92 kb) [file 12913_2019_4109_MOESM1_ESM.docx]

IBenC Questionnaire 2: characteristics of the home care organisations

This questionnaire is fourfold:

1. The structure of the organisation
2. Characteristics of the clients served by the organisation
3. Characteristics of the care giving staff
4. Organisational process

If you want to give more detailed answers or add any comments, space has been made for this purpose in the end of the questionnaire.

# Structure of the organization

1. Type of home care organisation

*My organisation provides:*

- Mainly nursing care
- Mainly social care
- Mixed nursing and social care

1. Establishment of the organisation

- The year in which your organisation was founded:

1. Ownership and type of organisation

*Indicate the type of ownership of your organisation:*

- Public/governmental/federal/municipal
- Private
  - For profit
  - Not for profit

1. Administrative structure

*Administratively, your organisation is*

- An independent organisation
- An organisation that is embedded in a larger care organisation

1. Director

*The director of the home care organisation is a:*

- Medical doctor
- Nurse
- Social worker
- Other, please specify:

1. Location

*The area, where your organisation provides care, is:*

- Rural
- Urban
- Mixed rural and urban

1. Size of service region

The region in which your organisation provides care in is approximately …..km^2^

*If your organisation is part of a chain of care organisations, or if your organisation is embedded in a larger care organisation, just give the size of the region relevant to that part of organisation that is included for the IBenC project.*

1. Organisation service provision structure

*In what types of setting does your organisation provide care? It is possible to indicate more options.*

- Community care
- Institutional care
- Acute care
- Rehabilitation care
- Mental health (including psychiatric care)
- Other, please specify

1. The provided care is fully paid out of:

|  | for all clients | for part of the clients | for none of the clients |
| --- | --- | --- | --- |
| Public/statutory health insurances |  |  |  |
| Private insurances |  |  |  |
| By clients’ own resources |  |  |  |
| A combination of public resources and clients’ resources |  |  |  |
| A combination of public resources and private insurances |  |  |  |
| A combination of private insurance and clients resources |  |  |  |
| A combination of all three |  |  |  |

1. Service provision

What type of services does your organisation provide? Check the box if at least one of the tasks is provided by your organisation. It is possible to indicate more options.

- IADL-services (e.g. cooking – meal preparation; shopping; ordinary housework like cleaning and laundry; meals on wheels; help with finances; transportation; medication management; …)
- ADL-services,(e.g. feeding; toileting; dressing upper or/and lower body; personal hygiene; bathing; positioning/repositioning)
- Specific medical care
- Other, please specify:

# Characteristics of the clients

1. Indicate the total number of clients in active care in your organisation as on January 1^ste^ 2015
   1. Total number of clients in active care:……
   2. Please specify the number of clients who are 65 years or older and make a division between those who need short term care and those who need long term care. Long term care means care needed for at least 6 months.
2. Number of clients aged who are 65 years or older: ……..clients
   1. Short term care: ….number of clients
   2. Long term care: …. number of clients
   3. As of January 1^st^ 2015, indicate how many clients from those who are 65 years or older, have dementia or display dementia symptoms?
      1. Dementia or displaying dementia symptoms: …….number of clients
      2. Of these, how many are formally diagnosed with dementia: …….number of clients
   - No data available
3. During the **previous calendar year 2014**, what was the number of **client visits?:**

*Count all the single client visits together. When a client receives 3 visits a day, this will count as 3 visits.*

1. The total number of client visits:
2. The number of visits for clients who are 65 years or older:

# Characteristics of the care giving staff

## Professions

1. How many employees are there at your workplace?

*If the organisation is part of a large company, the question only concerns the local workplace.*

- 1 (working alone)
- 2-4 employees
- 5-9 employees
- 10-19 employees
- 20-49 employees
- 50-99 employees
- 100-249 employees
- 250-499 employees
- 500 employees or more

1. Indicate the professions that are working in your organisation: (more options possible)

- Nurses and second level nurses
- Social workers
- Home health aides
- Management with a leading position
- Supportive administrative staff
- Other care professionals who provide care to the clients at home, please specify:…..

1. Indicate per profession the requested variables as of **January 1^st^ 2015.** Count all care professionals, those with a permanent and a temporary contract, also those who are on sick leave or on maternity or personal leave.
   1. **Nurses and second level nurses**

| Number and full time equivalent (FTE) per function |  |
| --- | --- |
| - **Second level nurses**    - Total FTE second level nurses:   - Total number of second level nurses:   - Full time:     - Total number of second level nurses: …     - Full time means …… hours working per week   - Part time:     - 80% or more (but lower than full time): …… second level nurses     - Between 50% and 79%: …… second level nurses     - Between 20% and 49%: …… second level nurses     - Lower than 20%: …… second level nurses - **Nurses**    - Total FTE of nurses:   - Total number of nurses:   - Full time:     - Total number of nurses: …     - Full time means …… hours working per week   - Part time:     - 80% or more (but lower than full time): …… nurses     - Between 50% and 79%: …… nurses     - Between 20% and 49%: …… nurses     - Lower than 20%: …… nurses |  |
| Number of nurses per age group (nurses + second level nurses) |  |
| 1. Younger than 20 years: …... persons 2. 20 – 24 years: ….. persons 3. 25 – 29 years: ….. persons 4. 30 – 34 years: ….. persons 5. 35 – 39 years: ….. persons 6. 40 – 44 years: ….. persons 7. 45 – 49 years: ….. persons 8. 50 – 54 years: ….. persons 9. 55 – 59 years: ….. persons 10. 60 - 64 years: ….. persons 11. 65 years or older: ….. of persons |  |
| Number of nurses per gender (nurses + second level nurses) |  |
| 1. Female: …. persons 2. Male: …. persons |  |
| Number of nurses per nationality (nurses + second level nurses) |  |
| 1. Belgian: …. persons (e.g. for Belgium, country specific) 2. European within the EU, other than Belgian: …. persons 3. European not within the EU: …. persons 4. Non-European: …. persons |  |
| Number of nurses per education level (nurses + second level nurses) |  |
| 1. Primary school: …. persons 2. Secondary school: …. persons 3. Higher than secondary school, but lower than bachelor degree: …. persons 4. Bachelor degree: …. persons 5. Masters: …. persons 6. PhD: …. persons 7. Other, please specify: …. persons |  |
| Number of nurses per seniority group. Seniority years mean the number of years that the nurse has worked **within your organisation**. (nurses + second level nurses) |  |
| 1. 0-5 years: …. persons 2. 5-10 years: …. persons 3. 10-20 years: …. persons 4. > 20 years: …. persons |  |
| Number of nurses per type of contract |  |
| 1. Permanent contract: …. persons 2. Temporary contract: …. persons | |
| Number of nurses who are on long-term sick leave on January 1^st^ 2015. Long-term sick leave means a sick leave of 30 days or more. Care professionals who are on maternity or on personal leave are not included. | |
| 1. Long-term sick leave: …. persons |  |

The countries within the European Union are: Austria, [Belgium](http://europa.eu/about-eu/countries/member-countries/belgium/index_en.htm), [Cyprus](http://europa.eu/about-eu/countries/member-countries/cyprus/index_en.htm), Czech Republic, [Denmark](http://europa.eu/about-eu/countries/member-countries/denmark/index_en.htm), [Estonia](http://europa.eu/about-eu/countries/member-countries/estonia/index_en.htm), [Finland](http://europa.eu/about-eu/countries/member-countries/finland/index_en.htm), France, [Germany](http://europa.eu/about-eu/countries/member-countries/germany/index_en.htm), [Greece](http://europa.eu/about-eu/countries/member-countries/greece/index_en.htm), [Hungary](http://europa.eu/about-eu/countries/member-countries/hungary/index_en.htm), [Ireland](http://europa.eu/about-eu/countries/member-countries/ireland/index_en.htm), [Italy](http://europa.eu/about-eu/countries/member-countries/italy/index_en.htm), [Latvia](http://europa.eu/about-eu/countries/member-countries/latvia/index_en.htm), [Lithuania](http://europa.eu/about-eu/countries/member-countries/lithuania/index_en.htm), [Luxembourg](http://europa.eu/about-eu/countries/member-countries/luxembourg/index_en.htm), [Malta](http://europa.eu/about-eu/countries/member-countries/malta/index_en.htm), [Netherlands](http://europa.eu/about-eu/countries/member-countries/netherlands/index_en.htm), [Poland](http://europa.eu/about-eu/countries/member-countries/poland/index_en.htm), [Portugal](http://europa.eu/about-eu/countries/member-countries/portugal/index_en.htm), [Romania](http://europa.eu/about-eu/countries/member-countries/romania/index_en.htm), [Slovakia](http://europa.eu/about-eu/countries/member-countries/slovakia/index_en.htm), [Slovenia](http://europa.eu/about-eu/countries/member-countries/slovenia/index_en.htm), [Spain](http://europa.eu/about-eu/countries/member-countries/spain/index_en.htm), [Sweden](http://europa.eu/about-eu/countries/member-countries/sweden/index_en.htm), [United Kingdom](http://europa.eu/about-eu/countries/member-countries/unitedkingdom/index_en.htm)

The European countries not within the European Union are: [Albania](http://ec.europa.eu/enlargement/countries/detailed-country-information/albania/index_en.htm), Andorra, Armenia, Belarus, [Bosnia and Herzegovina](http://ec.europa.eu/enlargement/countries/detailed-country-information/bosnia-herzegovina/index_en.htm), Georgia, [Iceland](http://ec.europa.eu/enlargement/countries/detailed-country-information/iceland/index_en.htm), Liechtenstein, ([Yugoslav Republic of) Macedonia](http://ec.europa.eu/enlargement/countries/detailed-country-information/fyrom/index_en.htm), Moldova, Monaco, [Montenegro](http://ec.europa.eu/enlargement/countries/detailed-country-information/montenegro/index_en.htm), Norway, Russia, San Marino, [Serbia](http://ec.europa.eu/enlargement/countries/detailed-country-information/serbia/index_en.htm), Switzerland, [Turkey](http://ec.europa.eu/enlargement/countries/detailed-country-information/turkey/index_en.htm), Ukraine, Vatican city

- 1. **Social workers**

| Number and full time equivalent (FTE) |
| --- |
| - - Total FTE of social workers:   - Total number of social workers:   - Full time:     - Total number of social workers: …     - Full time means ….. hours working per week   - Part time:     - 80% or more (but lower than full time): ……. social workers     - Between 50% and 79%: ……. social workers     - Between 20% and 49%: ……. social workers     - Lower than 20%: ……. social workers |
| Number of social workers per age group |
| 1. Younger than 20 years: ….. social workers 2. 20 – 24 years: ….. social workers 3. 25 – 29 years: ….. social workers 4. 30 – 34 years: ….. social workers 5. 35 – 39 years: ….. social workers 6. 40 – 44 years: ….. social workers 7. 45 – 49 years: ….. social workers 8. 50 – 54 years: ….. social workers 9. 55 – 59 years: ….. social workers 10. 60 - 64 years: ….. social workers 11. 65 years or older: ….. social workers |
| Number of social workers per gender |
| 1. Female: …. social workers 2. Male: …. social workers |
| Number of social workers per nationality |
| 1. Belgian: …. social workers (e.g. for Belgium, country specific) 2. European within the EU, other than Belgian: …. social workers 3. European not within the EU: …. social workers 4. Non-European: …. social workers |
| Number of social workers per education level |
| 1. Primary school: …. social workers 2. Secondary school: …. social workers 3. Higher than secondary school, but lower than bachelor degree: …. social workers 4. Bachelor degree: …. social workers 5. Masters: …. social workers 6. PhD: …. social workers 7. Other, please specify: …. social workers |
| Number of social workers per seniority group. Seniority years mean the number of years that the social worker has worked **within your organisation**. |
| 1. 0-5 years: …. social workers 2. 5-10 years: …. social workers 3. 10-20 years: …. social workers 4. > 20 years: …. social workers |
| Number of social workers per type of contract |
| 1. Permanent contract: …. social workers 2. Temporary contract: …. social workers |
| Number of social workers who are on long-term sick leave on January 1^st^ 2015. Long-term sick leave means a sick leave of 30 days or more. Care professionals who are on maternity or on personal leave are not included. |
| 1. Long-term sick leave: …. social workers |

- 1. **Home health aides**

| Number and full time equivalent (FTE) |
| --- |
| - - Total FTE of home health aides:   - Total number of home health aides :   - Full time:     - Total number of home health aides: …     - Full time means ….. hours working per week   - Part time:     - 80% or more (but lower than full time): …….home health aides     - Between 50% and 79%: …….home health aides     - Between 20% and 49%: …….home health aides     - Lower than 20%: …….home health aides |
| Number of home health aides per age group |
| 1. Younger than 20 years: …..home health aides 2. 20 – 24 years: …..home health aides 3. 25 – 29 years: …..home health aides 4. 30 – 34 years: …..home health aides 5. 35 – 39 years: …..home health aides 6. 40 – 44 years: …..home health aides 7. 45 – 49 years: …..home health aides 8. 50 – 54 years: …..home health aides 9. 55 – 59 years: …..home health aides 10. 60 - 64 years: …..home health aides 11. 65 years or older: …..home health aides |
| Number of home health aides per gender |
| 1. Female: …. home health aides 2. Male: …. home health aides |
| Number of home health aides per nationality |
| 1. Belgian: …. home health aides (e.g. for Belgium, country specific) 2. European within the EU, other than Belgian: …. home health aides 3. European not within the EU: …. home health aides 4. Non-European: …. home health aides |
| Number of home health aides per education level |
| 1. Primary school: …. home health aides 2. Secondary school: …. home health aides 3. Higher than secondary school, but lower than bachelor degree: …. home health aides 4. Bachelor degree: …. home health aides 5. Masters: …. home health aides 6. PhD: …. home health aides 7. Other, please specify: …. home health aides |
| Number of home health aides per seniority group. Seniority years mean the number of years that the home health aide has worked **within your organisation**. |
| 1. 0-5 years: …. home health aides 2. 5-10 years: …. home health aides 3. 10-20 years: …. home health aides 4. > 20 years: …. home health aides |
| Number of home health aides per type of contract |
| 1. Permanent contract: …. home health aides 2. Temporary contract: …. home health aides |
| Number of home health aides who are on long-term sick leave on January 1^st^ 2015. Long-term sick leave means a sick leave of 30 days or more. Care professionals who are on maternity or on personal leave are not included. |
| Long-term sick leave: …. home health aides |

- 1. **Management with a leading position**

| Number and full time equivalent (FTE) |
| --- |
| - - Total FTE of managers with a leading position:   - Total number of managers with a leading postion :   - Full time:     - Total number of managers with a leading position:….     - Full times means ….. hours working per week   - Part time:     - 80% or more (but lower than full time): ……. managers with a leading position     - Between 50% and 79%: ……. managers with a leading position     - Between 20% and 49%: ……. managers with a leading position     - Lower than 20%: ……. managers with a leading position |
| Number of managers with a leading position per age group |
| 1. Younger than 20 years: ….. managers with a leading position 2. 20 – 24 years: ….. managers with a leading position 3. 25 – 29 years: ….. managers with a leading position 4. 30 – 34 years: ….. managers with a leading position 5. 35 – 39 years: ….. managers with a leading position 6. 40 – 44 years: ….. managers with a leading position 7. 45 – 49 years: ….. managers with a leading position 8. 50 – 54 years: ….. managers with a leading position 9. 55 – 59 years: ….. managers with a leading position 10. 60 - 64 years: ….. managers with a leading position 11. 65 years or older: ….. managers with a leading position |
| Number of managers with a leading position per gender |
| 1. Female: …. managers with a leading position 2. Male: …. managers with a leading position |
| Number of managers with a leading position per nationality |
| 1. Belgian: …. managers with a leading position (e.g. for Belgium, country specific) 2. European within the EU, other than Belgian: …. managers with a leading position 3. European not within the EU: …. managers with a leading position 4. Non-European: …. managers with a leading position |
| Number of managers with a leading position per education level |
| 1. Primary school: …. managers with a leading position 2. Secondary school: …. f managers with a leading position 3. Higher than secondary school, but lower than bachelor degree: …. managers with a leading position 4. Bachelor degree: … managers with a leading position 5. Masters: …. managers with a leading position 6. PhD: …. managers with a leading position 7. Other, please specify: …. managers with a leading position |
| Number of managers with a leading position per seniority group. Seniority years mean the number of years that the manager has worked **within your organisation**. |
| 1. 0-5 years: … managers with a leading position 2. 5-10 years: …. managers with a leading position 3. 10-20 years: …. managers with a leading position 4. > 20 years: …. managers with a leading position |
| Number of managers with a leading position per type of contract |
| 1. Permanent contract: …. managers with a leading position 2. Temporary contract: …. managers with a leading position |
| Number of managers with a leading position who are on long-term sick leave on January 1^st^ 2015. Long-term sick leave means a sick leave of 30 days or more. Care professionals who are on maternity or on personal leave are not included. |
| 1. Long-term sick leave: …. managers with a leading position |

- 1. **Supportive administrative staff**

| Number and full time equivalent (FTE) |
| --- |
| - - Total FTE of administrative staff:   - Total number of administrative staff:   - Full time:     - Total number of supportive administrative staff:…     - Full time means ….. hours working per week   - Part time:     - 80% or more (but lower than full time): ……. supportive administrative staff     - Between 50% and 79%: ……. supportive administrative staff     - Between 20% and 49%: ……. supportive administrative staff     - Lower than 20%: ……. supportive administrative staff |
| Number of supportive administrative staff per age group |
| 1. Younger than 20 years: ….. supportive administrative staff 2. 20 – 24 years: ….. supportive administrative staff 3. 25 – 29 years: ….. supportive administrative staff 4. 30 – 34 years: ….. supportive administrative staff 5. 35 – 39 years: ….. supportive administrative staff 6. 40 – 44 years: ….. supportive administrative staff 7. 45 – 49 years: ….. supportive administrative staff 8. 50 – 54 years: ….. supportive administrative staff 9. 55 – 59 years: ….. supportive administrative staff 10. 60 - 64 years: ….. supportive administrative staff 11. 65 years or older: ….. supportive administrative staff |
| Number of supportive administrative staff per gender |
| 1. Female: …. supportive administrative staff 2. Male: …. supportive administrative staff |
| Number of supportive administrative staff per nationality |
| 1. Belgian: …. supportive administrative staff (e.g. for Belgium, country specific) 2. European within the EU, other than Belgian: …. supportive administrative staff 3. European not within the EU: …. supportive administrative staff 4. Non-European: …. supportive administrative staff |
| Number of supportive administrative staff per education level |
| 1. Primary school: …. supportive administrative staff 2. Secondary school: …. supportive administrative staff 3. Higher than secondary school, but lower than bachelor degree: …. supportive administrative staff 4. Bachelor degree: …. supportive administrative staff 5. Masters: …. supportive administrative staff 6. PhD: …. supportive administrative staff 7. Other, please specify: …. supportive administrative staff |
| Number of supportive administrative staff per seniority group. Seniority years mean the number of years that the person with an administrative function has worked **within your organisation**. |
| 1. 0-5 years: ….supportive administrative staff 2. 5-10 years: …. supportive administrative staff 3. 10-20 years: …. supportive administrative staff 4. > 20 years: …. supportive administrative staff |
| Number of supportive administrative staff per type of contract |
| 1. Permanent contract: …. supportive administrative staff 2. Temporary contract: …. supportive administrative staff |
| Number of supportive administrative staff who are on long-term sick leave on January 1^st^ 2015. Long-term sick leave means a sick leave of 30 days or more. Care professionals who are on maternity or on personal leave are not included. |
| Long-term sick leave: …. supportive administrative staff |

- 1. **Other care professionals who provide care to the clients at home, specify**

| Number and full time equivalent (FTE) |
| --- |
| - - Total FTE of other care professionals who provide care to the clients at home :   - Total number of other care professionals who provide care to the clients at home :   - Full time:     - Total number of other care professionals who provide care to the clients at home: ….     - Full time means ….. hours working per week   - Part time:     - 80% or more (but lower than full time): ……. other care professionals who provide care to the clients at home     - Between 50% and 79%: ……. other care professionals who provide care to the clients at home     - Between 20% and 49%: ……. other care professionals who provide care to the clients at home     - Lower than 20%: ……. other care professionals who provide care to the clients at home |
| Number of ‘others’ per age group |
| 1. Younger than 20 years: ….. persons 2. 20 – 24 years: ….. persons 3. 25 – 29 years: ….. persons 4. 30 – 34 years: ….. persons 5. 35 – 39 years: ….. persons 6. 40 – 44 years: ….. persons 7. 45 – 49 years: ….. persons 8. 50 – 54 years: …..persons 9. 55 – 59 years: ….. persons 10. 60 - 64 years: ….. persons 11. 65 years or older: ….. persons |
| Number of ‘others’ per gender |
| 1. Female: …. persons 2. Male: …. persons |
| Number of ‘other’ per nationality |
| 1. Belgian: …. persons (e.g. for Belgium, country specific) 2. European within the EU, other than Belgian: …. persons 3. European not within the EU: …. persons 4. Non-European: …. persons |
| Number of ‘others’ per education level |
| 1. Primary school: …. persons 2. Secondary school: …. persons 3. Higher than secondary school, but lower than bachelor degree: …. persons 4. Bachelor degree: …. persons 5. Masters: …. persons 6. PhD: …. persons 7. Other, please specify: …. persons |
| Number of ‘others’ per seniority group. Seniority years mean the number of years that the ‘others’ have worked **within your organisation**. |
| 1. 0-5 years: …. persons 2. 5-10 years: …. persons 3. 10-20 years: …. persons 4. > 20 years: …. persons |
| Number of ‘others’ per type of contract |
| 1. Permanent contract: …. persons 2. Temporary contract: …. persons |
| Number of ‘others’ who are on long-term sick leave on January 1^st^ 2015. Long-term sick leave means a sick leave of 30 days or more. Care professionals who are on maternity or on personal leave are not included. |
| Long-term sick leave: …. persons |

1. As for the situation on the 1st of January 2015, does your organisation work with volunteers?
   - No
   - Yes, on January 1th 20124 we had…. volunteers who working in our organisation.
2. Does your organisation have specialised care professionals?
   - No
   - Yes, specialised in the following domains (more options possible)
     1. Dementia
     2. Psychiatric disorders
     3. Palliative care
     4. Specific medical condition, please specify…
     5. Other, please specify…
3. Does your organisation makes use of a flex pool with temporary workers
   - No
   - Yes, only during the weekends
   - Yes, only during the holidays
   - Yes, when it is needed

## Turnover

Indicate the **number** of care professionals who left the organisation **during the last calendar year (2014)**.

1. Second level nurses:

- …… number

1. Nurses:
   - …… number
2. Social workers:
   - …… number
3. Home health aides:
   - …… number
4. Management with a leading position:
   - …… number
5. Supportive administrative staff:
   - …… number
6. Other care professionals who provide care to the clients at home, specify
   - …… number

## Education and training

1. Indicate the **total number of hours of training** received during the **previous calendar year (2014)**, by the care professionals who had a contract with your organisation on the 1st of January 2015 (see question A3). Count all care giving staff members, those with a permanent as well as those with a temporary contract, and those who are on sick leave or on maternity or personal leave.

Internal meetings and multidisciplinary meetings are not counted as training.

1. Second level nurses:
   - …… number of hours
2. Nurses:
   - …… number of hours
3. Social workers:
   - …… number of hours
4. Home health aides
   - …… number of hours
5. Management with a leading position
   - …… number of hours
6. Supportive administrative staff
   - …… number of hours
7. Other care professionals who provide care to the clients at home, specify
   - …… number of hours
8. Indicate whether or not the organisation is **legally required** to provide the opportunity for staff to undertake additional training and education.

Also indicate the maximal number of hours per year that the **organisation facilitated** staff to undertake additional training and education. (internal or external education or training)

- 1. **Second level nurses**

1. We are legally required to provide additional training and education to second level nurses
   - Yes
   - No
2. For second level nurses, the maximum number of training hours offered by the organisation is *per 1 FTE second level nurse*  ….. hours a year.
   1. **Nurses**
3. We are legally required to provide additional training and education to nurses
   - Yes
   - No
4. For nurses, the maximum number of training hours offered by the organisation is *per*  *1 FTE nurse* ….. hours a year.
   1. **Social workers**
5. We are legally required to provide additional training and education to social workers
   - Yes
   - No
6. For social workers, the maximum number of training hours offered by the organisation is *per 1 FTE social worker* ….. hours a year.
   1. **Home health aides**
7. We are legally required to provide additional training and education to home health aides
   - Yes
   - No
8. For home health aides, the maximum number of training hours offered by the organisation is *per*  *1 FTE home health aide* ….. hours a year.
   1. **Management with a leading position**
9. We are legally required to provide additional training and education to managers
   - Yes
   - No
10. For managers, the maximum number of training hours offered by the organisation is *per 1 FTE manager with a leading position* ….. hours a year.
    1. **Supportive administrative staff**
11. We are legally required to provide additional training and education to the administrative staff
    - Yes
    - No
12. For administrative staff, the maximum number of training hours offered by the organisation is *per 1 FTE supportive administrative staff* ….. hours a year.
    1. **Other care professionals who provide care to the clients at home, please specify:……..**
13. We are legally required to provide additional training and education to ‘others’
    - Yes
    - No
14. For staff with other functions as previously mentioned, the maximum number of training hours offered by the organisation is *per 1 FTE ‘other care professional who provide care to the client’* ….. hours a year.

## Benefits

1. Please indicate which benefits exist in your organisation. Also indicate whether the benefit exists for the care professionals, the managers or for both and whether the benefit is required by law or is additional offered by your organisation.

| **Benefits** | **Benefit exits for** | **Benefit is** |
| --- | --- | --- |
| Induction or in-services training for new staff | the care professionals  the managers  both | required by law  additional offered by our organisation |
| Possibility of having unpaid or paid study leave | the care professionals  the managers  both | required by law  additional offered by our organisation |
| Opportunity for promotion | the care professionals  the managers  both | required by law  additional offered by our organisation |
| Basic health insurance | the care professionals  the managers  both | required by law  additional offered by our organisation |
| Health insurance on top of the legal mandatory insurance, partially or fully paid for the staff by the organisation (e.g. hospitalisation insurance for the care professional) | the care professionals  the managers  both | required by law  additional offered by our organisation |
| Health insurance on top of the legal mandatory insurance, partially or fully paid for the staff and his or her family by the organisation (e.g. hospitalisation insurance for the care professional and his or her family) | the care professionals  the managers  both | required by law  additional offered by our organisation |
| Early retirement | the care professionals  the managers  both | required by law  additional offered by our organisation |
| Paid overtime | the care professionals  the managers  both | required by law  additional offered by our organisation |
| Paid personal days, e.g. a paid personal day for staying at home with your sick child | the care professionals  the managers  both | required by law  additional offered by our organisation |
| Paid vacation days | the care professionals  the managers  both | required by law  additional offered by our organisation |
| Extra pay for evening or night shifts | the care professionals  the managers  both | required by law  additional offered by our organisation |
| Extra pay for working on weekend days | the care professionals  the managers  both | required by law  additional offered by our organisation |
| Extra pay for working on public holidays | the care professionals  the managers  both | required by law  additional offered by our organisation |
| Performance bonus | the care professionals  the managers  both | required by law  additional offered by our organisation |
| Fee per kilometer for the use of a personal car | the care professionals  the managers  both | required by law  additional offered by our organisation |
| A car provided by the organisation | the care professionals  the managers  both | required by law  additional offered by our organisation |
| Meal allowances or free meals | the care professionals  the managers  both | required by law  additional offered by our organisation |
| Kindergarten that belongs to the organisation | the care professionals  the managers  both | required by law  additional offered by our organisation |
| Other, namely: | the care professionals  the managers  both | required by law  additional offered by our organisation |

1. The working conditions in the organisation are arranged according to a collective labour agreement

- Yes
- No

1. Indicate for each profession the average **hourly gross wage** in € for a **full time** employee with **5 years of work experience** in this specific setting:
2. Second level nurses: ………€
3. Nurses: ………€
4. Social workers: ………€
5. Home health aides: ………€
6. Managers with a leading position: ………€
7. Supportive administrative staff: ………€
8. Other care professionals, who provide care to the clients at home, please specify: ………€

# Organisational Process

1. Indicate the length of each shift by the number of hours:

**On weekdays**

- Day shift:
- Evening shift:
- Night shift:
- A split shift (when a shift is interrupted for more than one hour, lunch time not included):
  - Part one of the split shift:
  - Part two of the split shift:
- Other, please specify

**On weekend days**

- Day shift:
- Evening shift:
- Night shift:
- A split shift (when a shift is interrupted for more than one hour, lunch time not included):
  - Part one of the split shift:
  - Part two of the split shift:
- Other, please specify:

1. By whom are the shifts scheduled?
   - By a central back office
   - By the team manager
   - By the employee him/herself
   - Other, please specify…..
2. Were there cutbacks in your organisation during the calendar year 2014: (more options possible)
   - No
   - Yes, in terms of number of FTE staffing
   - Yes, in terms of benefits
   - Yes, in terms of payment
   - Yes, in terms of purchase of materials to provide care to the clients
   - Yes, other
3. Is there a written mission and vision in your organisation:
   - Yes
   - No

## Professional on call

1. *In your organisation is a care professional on call?* (more options possible)

- Yes, always
- Yes, during the morning shift
- Yes, during the evening shift
- Yes, during the night shift
- Yes, during the weekends
- Yes, other. Specify:
- No, never

1. *Is there a general practitioner on call? (a GP whether or not connected with the organisation)*

- Yes, 24/7
- Yes, only on weekdays
- Yes, only on weekend days
- No, there is no general practitioner on call

1. *The general practitioner on call is:* (more options possible)

- Employed by the organisation
- Always the general practitioner of the client
- A general practitioner on shift in the region
- There is no general practitioner on call

1. *After a call, the general practitioner reacts*

- Within 10 minutes
- Between 10-30 minutes
- After 30 minutes
- The GP does not react, after a call

1. *After a call, the general practitioner can be at the home of the client*

- Within 30 minutes
- After 30 minutes
- The GP does not come to the client’s home, after a call

## Care management

1. In our organisation, case management is a part of our service.
   - No
   - Yes
      Please describe the kind of case management your organisation provides:
2. In our organisation, disease management is a part of our service.
   - No
   - Yes
      Please describe the kind of disease management your organisation provides:

## Referral and transfer

1. Does your organisation have a single entry point for the referral of potential clients?

- Yes
- No

1. Who can refer clients to your organisation? (more options possible)

- The general practitioner
- Social services
- Hospitals
- A client or family member
- All
- Other, please specify …

1. When a client is referred to your organisation, is eligibility criteria checked to determine if the client can be admitted in the organisation?

- No
- Yes
  - - If so, is a standardised form used?
  - Yes
  - No
    - The following criteria are checked: (more options possible)
  - Physical function level
  - Cognitive function level
  - Presence of psychiatric diseases
  - Family situation
  - Means tested
  - Medical prescriptions
- Who is consulted in gathering the information to enable a decision to be made on the eligibility criteria? (more options possible)
  - Second level nurses
  - Nurses
- Home care aides
- Social workers
- General Practitioner
- Team supervisor
- Manager of the organisation
- Director of the organisation
- Physiotherapist
- Occupational therapist
- Psychologist
- Clients
- Family / informal carer of the client
- Other, please specify:

1. When a client is temporary discharged from your organisation, e.g. because of an hospital admission, your organisation provides a transfer document

- Yes, always
- Yes, mostly
- Yes, sometimes
- No, we do not use transfer documents

1. When a client is permanently discharged from your organisation, e.g. because of an admission to a long care term facility, your organisation provides a transfer document

- Yes, always
- Yes, mostly
- Yes, sometimes
- No, we do not use transfer documents

## The client file and geriatric assessments

1. Has your organisation already used an interRAI instrument before using it for this specific IBenC project (e.g. MDS, contact assessment, interRAI Home care, …)?
   - yes
   - no
2. Before using the interRAI instruments for this specific IBenC project, are a single assessment instrument or series of instruments used in your care organisation to assess geriatric symptoms?

Please indicate for every domain whether there is a single or series of assessment instruments used in the organisation.

| **Domain** | **Assessed** |
| --- | --- |
| ADL | No Yes |
| IADL | No Yes |
| Depression | No Yes |
| Cognition | No Yes |
| Social engagement | No Yes |
| Health status | No Yes |
| Hearing/vision | No Yes |
| Dietary status | No Yes |
| Informal care burden | No Yes |

1. Indicate which professionals / persons are involved by the assessments. By involved we mean consulted on data about the client or filled out some items of the assessment instrument. (more options possible)

- Second level nurses
- Nurses
- Home care aides
- Social workers
- General Practitioner
- Team supervisor
- Manager of the organisation
- Director of the organisation
- Physiotherapist
- Occupational therapist
- Psychologist
- Clients
- Family / informal carer of the client
- Other, specify

1. How frequently are geriatric assessments performed (whether or not on the base of a geriatric assessment instrument)? Please check if applicable.

|  | On admission only | On admission and any time when significant change in status | On admission and at regular time intervals | Other, specify |
| --- | --- | --- | --- | --- |
| ADL |  |  | every …… months | ………….. |
| IADL |  |  | every …… months | ………….. |
| Depression |  |  | every …… months | ………….. |
| Cognition |  |  | every …… months | ………….. |
| Social engagement |  |  | every …… months | ………….. |
| Health status |  |  | every …… months | ………….. |
| Hearing/vision |  |  | every …… months | ………….. |
| Dietary status |  |  | every …… months | ………….. |
| Informal care burden |  |  | every …… months | ………….. |

1. After a geriatric assessment (whether or not on the basis of a geriatric assessment instrument), a meeting to discuss the specific care planning is organised

- No, we do not discuss the geriatric assessments in teams
- Yes, after the admission assessment only
- Yes, after the admission assessment and any time when there is a significant change in the status
- Yes, after the admission assessment and at regular times intervals, namely every…….months for each client
- Other, please specify

1. Is there a multidisciplinary team discussing the outcomes of the geriatric assessments during these meetings?

- No
- Yes
- How many people on average are present during these meetings:
- Indicate the professions / person who are usually present during those meetings: (more options possible)
  - Second level nurses
  - Nurses
  - Home care aides
  - Social workers
  - General Practitioner
  - Team supervisor
  - Manager of the organisation
  - Director of the organisation
  - Physiotherapist
  - Occupational therapist
  - Psychologist
  - Clients
  - Family / informal carer of the client
  - Other, please specify:

1. Is there a policy to discuss the care plan with the clients and his or her family/informal carer?
   - No
   - Yes 🡪 every ….. months
2. Is there a policy to involve the family/informal carer actively in the care for the client?
   - No
   - Yes
3. In your organisation the client file and/or the geriatric assessments are:

- On paper
- Digital
- On paper and digital
- Available in the organisation only
- Available at the client’s home only
- Available in the organisation and at the client’s home

## Meetings

1. **Management meetings**

Definition: a management meeting is a meeting with the managers of the organisation to discuss the overall operation of the organisation.

- How often is a management meeting organized in your organisation?
- Daily
- Several times a week
- Weekly
- Two weekly
- Monthly
- Between two monthly and six monthly
- 6 monthly
- Less than 6 monthly
- Never
- What is the average duration of a management meeting: ……minutes
- How many people on average are present during an management meeting:
- Who are usually present during an management meeting: (more options possible)
- Second level nurses
- Nurses
- Home care aides
- Social workers
- General Practitioner
- Team supervisor
- Manager of the organisation
- Director of the organisation
- Physiotherapist
- Occupational therapist
- Psychologist
- Clients
- Family / informal carer of the client
- Other, specify

1. **Internal team organisation meetings**

Definition:an internal team organisation meeting means a meeting with the home care professionals of the same organisation to inform and to discuss with the care professionals the overall operation of the organisation.

- How often is an internal team organisation meeting organized in your organisation:
- Daily
- Several times a week
- Weekly
- Two weekly
- Monthly
- Between two monthly and six monthly
- 6 monthly
- Less than 6 monthly
- Never
- What is the average duration of an internal team meeting: ……minutes
- How many people on average are present during an internal team organisation meeting:
- Who are usually present during an internal team organisation meeting: (more options possible)
- Second level nurses
- Nurses
- Home care aides
- Social workers
- General Practitioner
- Team supervisor
- Manager of the organisation
- Director of the organisation
- Physiotherapist
- Occupational therapist
- Psychologist
- Clients
- Family / informal carer of the client
- Other, please specify:

1. **Clients’ briefing in team**

Definition: a clients’ briefing means a meeting with the home care professionals of the same organisation to transfer information about the clients.

- How often is a clients’ briefing organized in your organisation:
- Daily
- Several times a week
- Weekly
- Two weekly
- Monthly
- Between two monthly and six monthly
- 6 monthly
- Less than 6 monthly
- Never
- What is the average duration of a clients’ briefing: ……minutes
- How many people on average are present during a clients’ briefing:
- Who are usually present during an clients’ briefing: (more options possible)
- Second level nurses
- Nurses
- Home care aides
- Social workers
- General Practitioner
- Team supervisor
- Manager of the organisation
- Director of the organisation
- Physiotherapist
- Occupational therapist
- Psychologist
- Clients
- Family / informal carer of the client
- Other, please specify:

## Accountability

1. Are there annual reports on the performance (e.g. financial status, care policy during last year, structure care professionals …) of the organisation available.
   - Yes, publicly
   - Yes, for the government/federal authorities
   - Yes, for insurers
   - Yes, only for the organisation
   - No, not available
2. Is the quality of care in the organisation measured?

- No, our organisation does not assess quality of care
- Yes, our organisation assess quality of care
  - How often does your organisation assess quality of care?
    - More than six-monthly
    - Six-monthly
    - Every year
    - Each two years
    - Less than two-yearly, please specify:
  - On what basis does your organisation assess quality of care?
    - On a voluntary basis
    - Requirement due to legislation
    - Both
  - Are the results of the quality of care assessment available:
    - Yes, the results are publicly available
    - Yes, the results are available for the government/federal authority
    - Yes, the results are available for the insurers
    - No, the results are only available for the organisation, for internal use
  - Is the quality of care assessed using a standardised instrument/document?
    - Yes, please specify
    - No

1. Is client satisfaction on the received care of your organisation measured?
   - No, our organisation does not assess client satisfaction
   - Yes, our organisation assesses client satisfaction
     - How often does your organisation assess client satisfaction?
       - More than six-monthly
       - Six-monthly
       - Every year
       - Each two years
       - Less than two-yearly, please specify:
     - On which basis does your organisation assess client satisfaction?
       - On a voluntary basis
       - Requirement due to legislation
       - Both
     - Are the results of the client satisfaction measurement available:
       - Yes, the results are publicly available
       - Yes, the results are available for the government
       - Yes, the results are available for the insurers
       - No, the results are only available for the organisation, for internal use
     - Is client satisfaction assessed using a standardised instrument / document?
       - Yes
       - No

# Additional comments

If you feel that certain aspects that define your organisation are not sufficiently addressed in the questionnaire, please describe them here:

Other comments:
