# Supplementary material for: Development of a novel benchmark method to identify and characterize best practices in home care across six European countries: design, baseline, and rationale of the IBenC project
Source: BMC Health Serv Res. 2019 May 15;19:310. doi: 10.1186/s12913-019-4109-y (PMC6521361; doi:10.1186/s12913-019-4109-y)
Supplement: Supplementary file 2 — Appendix 2. IBenC project: Questionnaire 1- characteristics of the care giving staff. Questionnaire developed within the IBenC project to assess characteristics of professional caregivers working in the participating home care organizations. (DOCX 72 kb) [file 12913_2019_4109_MOESM2_ESM.docx]

IBenC Questionnaire 1- characteristics of the care giving staff

To fill out by the care professionals

Dear Madam/Sir,

Thank you very much for your participation in the IBenC study. In this project we study which characteristics of home care organizations might contribute to good quality and affordable home care, this also includes home care staff.

This questionnaire is developed as a tool to measure characteristics home care staff in Europe.

The questions concern your conditions and your opinions. You should therefore avoid “help” from others. Please note that there are no correct or wrong answers. It is important for the quality of the study that you answer all the questions.

Your participation is voluntary. If you are not willing to fill in the questionnaire this does not influence your work situation in any way.

Your answers are being processed anonymously. The results are calculated for groups so that the individual responses cannot be identified. It is the home care organization that is being studied. Not the individual employees.

It takes about 30 minutes to fill out the questionnaire. You answer the questions by ticking the right box. In some of the questions you are asked to write a number or a few words. At the end of the questionnaire, you can write down any comments or more detailed answers on questions if you want to.

By reading the statement below and checking the boxes, you agree in participation in the study.

Good luck and thank you in advance!

[Text for online informed consent IBenC for staff of participating care organisations]

**Informed Consent IBENC**

**Identifying best practices for care-dependent elderly by Benchmarking Costs and outcomes of community care**

By checking the box at the bottom of this page I declare to participate in the IBenC project. I hereby declare that:

- I have been informed sufficiently and satisfactory about the aims and the content of the IBenC project.
- I have received the written information about the project.
- I have had the opportunity to ask questions about the study. My questions have been answered to my satisfaction.
- I know that my participation is voluntarily and that I can and are allowed to withdraw my cooperation to the study at any time.
- I understand that as part of the study data are being collected and processed. I voluntarily agree with this.
- I know that my data will be stored and processed anonymously, and that nobody who is not a member of the IBenC research team, besides myself, has access to these data.
- I give consent to store my data after the project ends.
- I consent to participate in the IBenC project.

**Please check to box to give your consent and start the questionnaire.**

# Function at the organisation

For the IBenC researcher: Depending on this answer the next questionnaire will open:

1. The questionnaire for the caregivers: nurse/second level nurse; social worker, Home health aide; other
2. The questionnaire for the managers
3. The questionnaire for the administrative staff
4. Indicate the function at your current employer.

- Nurse / second level nurse
- Social worker
- Home health aide
- Manager with a leading position
- Supportive administrative staff
- Other, please specify:

# Social conditions, background and living habits

1. What is your highest level of education?

- primary school
- secondary school
- higher than secondary school, but lower than bachelor
- bachelor
- master
- PhD
- Other, please specify

1. What is the total number of months you have received additional vocational education or training after finishing your school career (exclude conferences)?
2. Indicate in which age group you are in:
   - Younger than 20 years
   - 20 – 24 years
   - 25 – 29 years
   - 30 – 34 years
   - 35 – 39 years
   - 40 – 44 years
   - 45 – 49 years
   - 50 – 54 years
   - 55 – 59 years
   - 60 - 64 years
   - 65 years or older
3. Gender:

- female
- male

1. Please indicate your nationality (e.g. for Belgium):

- Belgian
- Other European within the EU
- European not within the EU
- Non-European

1. Please indicate your current civil status:

- never married
- married / partner
- widowed
- separated / divorced

1. Please indicate your living arrangement

- alone
- with spouse/ partner and/or children
- with other

1. How many children are part of your household?

*Please note that this only concerns children that are still living at home. Children who no longer live at home, are not included.*

- - None
  - 1
  - 2
  - 3
  - 4 or more

1. Who is primarily responsible for the care of children?
   - myself together with my partner
   - myself alone
   - my partner alone
   - co-parenting/joint custody
   - somebody else
   - not applicable
2. Are you taking care of disabled, ill or elderly people in your private life?
   - Yes
   - No
3. Please indicate in which category of following scales of income your monthly average family net income is located? This is the summation of all household incomes of all family members together.

- 249,99€ per month or lower
- 250,00€ - 499,99€ per month
- 500,00€ - 999,99€ per month
- 1000,00€ - 1499,99€ per month
- 1500,00€ - 1999,99€ per month
- 2000,00€ - 2499,99€ per month
- 2500,00€ - 2999,99€ per month
- 3000,00€ - 3999,99€ per month
- 4000,00€ - 4999,99€ per month
- € 5000,00 per month or more

1. Do you have enough money to meet your needs?
   - Not at all
   - A little
   - Moderately
   - Mostly
   - Completely
2. To what extent do you agree with the following statements about your social support outside the workplace?

|  |  | Totally agree | Some-what agree | Neither agree, nor disagree | Some-what disagree | Totally disagree | Not appli-cable |
| --- | --- | --- | --- | --- | --- | --- | --- |
| 14.1 | I can rely on my spouse when things are tough at work | □ | □ | □ | □ | □ | □ |
| 14.2 | I can rely on my friends when things are tough at work | □ | □ | □ | □ | □ | □ |
| 14.3 | I can rely on my other relatives when things are tough at work | □ | □ | □ | □ | □ | □ |
| 14.4 | My spouse is willing to listen to my personal problems | □ | □ | □ | □ | □ | □ |
| 14.5 | My friends are willing to listen to my personal problems | □ | □ | □ | □ | □ | □ |
| 14.6 | My other relatives are willing to listen to my personal problems | □ | □ | □ | □ | □ | □ |

# Health and well-being

The next questions are about your health and well-being

1. **The following questions are about your *own* health and well-being. Please do not try to distinguish between symptoms that are caused by work and symptoms that are due to other causes. The task is to describe how you are in general.**

|  | **during the last four weeks** | Excellent | Very good | Good | Fair | Poor |
| --- | --- | --- | --- | --- | --- | --- |
| C8 | In general, would you say your health is | □ | □ | □ | □ | □ |

|  | **during the last four weeks** | All the time | A large part of the time | Part of the time | A small part of the time | Not at all |
| --- | --- | --- | --- | --- | --- | --- |
| C10.16 | How often have you been irritable? | □ | □ | □ | □ | □ |
| C 10. 30 | How often have you been stressed? | □ | □ | □ | □ | □ |

|  |  | Always | Often | Some-times | Seldom | Never/ almost never |
| --- | --- | --- | --- | --- | --- | --- |
| CBI 1 | How often do you feel tired? | □ | □ | □ | □ | □ |
| CBI 2 | How often are you physically exhausted? | □ | □ | □ | □ | □ |
| CBI 3 | How often are you emotionally exhausted? | □ | □ | □ | □ | □ |
| CBI4 | How often do you think: 'I can't take it anymore?' | □ | □ | □ | □ | □ |
| CBI 5 | How often do you feel worn out? | □ | □ | □ | □ | □ |
| CBI 6 | How often do you feel weak and susceptible? | □ | □ | □ | □ | □ |

# Personal job characteristics

1. How many years and months do you work for this employer (or as self-employed)?

…..years and …..months

1. Do you have a superior?
   - Yes
   - No
2. Which type of contract do you have:
   - Permanent contract
   - Temporary contract
   - I work as a self-employed
   - No contract
3. What is your current employment situation?

- Full time work 🡪 I work ….. hours/ week
- Part-time
  - - 80% or more (but lower than full time)🡪 I work ….. hours/ week
    - Between 50% and 79%🡪 I work ….. hours/ week
    - Between 20% and 49% 🡪 I work ….. hours/ week
    - Lower than 20% 🡪 I work ….. hours/ week

1. If you work part-time: The reason that you work part-time

- Is a forced choice
- Is a voluntary choice

1. How is your work shift arrangement

- Fixed day work (primarily between 6h and 18h)
- Fixed evening work (primarily between 15h and 24h)
- Fixed night work (primarily between 22h and 6h)
- Variable working hours without night work
- Variable working hours with night work

1. How many hours does an average shift last? ….. hours
2. How many years of work experience do you have in elderly care? …..years
3. How many years of work experience do you have in home care? ……years
4. Do you currently work for pay in a similar job for more than one employer?

- Yes
- No

1. How many days were you on sick leave in the previous calendar year (2013)?

- I was not on sick leave in 2013
- One day to one week
- More than one week, but less than one month
- One – two months
- Two – six months
- More than six months

1. Decision making

27.1 To what extend do you feel the following professionals in your organisation decide upon the organisational goals (e.g. income and expenses of the organisation, case load, care approach, change trajectories, etc.)

|  | To a very large extent | To a large extent | Somewhat | To a small extent | To a very small extent | Professional not present in our organisation |
| --- | --- | --- | --- | --- | --- | --- |
| Board of directors / director | □ | □ | □ | □ | □ | □ |
| Team leaders | □ | □ | □ | □ | □ | □ |
| Quality mangers / other mangers | □ | □ | □ | □ | □ | □ |
| Whole team | □ | □ | □ | □ | □ | □ |
| myself | □ | □ | □ | □ | □ | □ |
| Other: | □ | □ | □ | □ | □ | □ |

27.2 To what extend do you feel the following professionals in your organisation decide upon decisions on the work methods and work processes of the organisation (e.g. delineating the care plan, duration of client visits, care plan discussions, care tasks to be performed etc.)

|  | to a very large extent | To a large extent | Somewhat | To a small extent | To a very small extent | Professional not present in our organisation |
| --- | --- | --- | --- | --- | --- | --- |
| Board of directors / director | □ | □ | □ | □ | □ | □ |
| Team leaders | □ | □ | □ | □ | □ | □ |
| Quality mangers / other mangers | □ | □ | □ | □ | □ | □ |
| Whole team | □ | □ | □ | □ | □ | □ |
| Myself | □ | □ | □ | □ | □ | □ |
| Other | □ | □ | □ | □ | □ | □ |

1. How many employees are working together with you in a team? *Think of the team in which you work within your own organisation.*

- I do not work in a team
- The team wherein I work counts:
- ….. Supervisors / managers
- ….. Other colleagues

1. Case load (following items are required only for the care professionals)

|  |  | No client | 1-5 clients | 6-10 clients | 11-15 clients | 16-20 clients | > 20 clients |
| --- | --- | --- | --- | --- | --- | --- | --- |
| 29a | For how many clients you provide care on an average shift in the week? | □ | □ | □ | □ | □ | □ |
| 29b | Of those clients, how many are 65 years or older | □ | □ | □ | □ | □ | □ |
| 29c | Of the clients, who are 65 years or older, how many have dementia or have displaying dementia symptoms | □ | □ | □ | □ | □ | □ |
| 29d | Of the clients, who are 65 years or older, how many are **only** ADL-depended  (depended for e.g. feeding; toileting; dressing upper or/and lower body; personal hygiene; bathing; positioning/repositioning) | □ | □ | □ | □ | □ | □ |
| 29e | Of the clients, who are 65 years or older, how many are **only** IADL-depended (depended for e.g. cooking – meal preparation; shopping; ordinary housework like cleaning and laundry; meals on wheels; help with finances; transportation; medication management; …) | □ | □ | □ | □ | □ | □ |
| 29f | Of the clients, who are 65 years or older, how many are ADL **and** IADL depended | □ | □ | □ | □ | □ | □ |

1. To what extent are you familiar with the care and welfare services that are available in the area you work in?

- to a very large extent
- to a large extent
- somewhat
- to a small extent
- to a very small extent

1. How many minutes do you spend on average travelling between clients during a shift?

…..minutes

# Psychosocial factors at work

The following questions are about your work environment. Some of the questions may fit better to your work than others, but please answer all questions.

|  |  | Always | Often | Sometimes | Seldom | Never/hardly never |
| --- | --- | --- | --- | --- | --- | --- |
| C32.3 | Does your work put you in emotionally disturbing situations? | □ | □ | □ | □ | □ |
| CBI10 | Do you feel worn out at the end of the working day? | □ | □ | □ | □ | □ |
| C32.4 | Do you have a large degree of influence concerning your work? | □ | □ | □ | □ | □ |
| C32.8 | Do you have to relate to other people’s personal problems as part of your work? | □ | □ | □ | □ | □ |
| CBI11 | Are you exhausted in the morning at the thought of another day at work? | □ | □ | □ | □ | □ |
| C32.14 | Do you get behind with your work? | □ | □ | □ | □ | □ |
| C32.23 | Do you have enough time for your work tasks? | □ | □ | □ | □ | □ |
| CBI12 | Do you feel that every working hour is tiring for you? | □ | □ | □ | □ | □ |
| C32.24 | Can you influence the amount of work assigned to you? | □ | □ | □ | □ | □ |
| CBI13 | Do you have enough energy for family and friends during leisure time? | □ | □ | □ | □ | □ |
| C32.29 | Can you decide when to take a break? | □ | □ | □ | □ | □ |
| IBenC 1 | Do you have to do overtime? | □ | □ | □ | □ | □ |
| CBI18 | Are you tired of working with clients? | □ | □ | □ | □ | □ |
| C32.31 | Can you leave your work to have a chat with your colleague? | □ | □ | □ | □ | □ |
| CBI19 | Do you sometimes wonder how long you will be able to continue working with clients? | □ | □ | □ | □ | □ |
| C47.1 | How often is your nearest superior willing to listen to your problems at work? | □ | □ | □ | □ | □ |
| C47.3 | How often does your nearest superior talk with you about how well you carry out your work? | □ | □ | □ | □ | □ |

|  |  | Never | Sometimes | Often | Always |
| --- | --- | --- | --- | --- | --- |
| PWS1 | My job requires fast and sustained physical efforts | □ | □ | □ | □ |
| PWS2 | My job involves repetitive movements of the same part of my body (muscle, tendon, joint,…) | □ | □ | □ | □ |
| PWS3 | During my work I have to move or lift heavy loads | □ | □ | □ | □ |
| PWS4 | I have to work for long periods in awkward postures | □ | □ | □ | □ |

1. **To what extent, if at all, each of the following is a rewarding part of your job:**

|  |  | not at all rewarding | somewhat rewarding | very rewarding | extremely rewarding | does not apply to my job |
| --- | --- | --- | --- | --- | --- | --- |
| JRQ1 | Helping others is… | □ | □ | □ | □ | □ |
| JRQ2 | Making a difference in other people's lives is… | □ | □ | □ | □ | □ |
| JRQ3 | Being needed by others is… | □ | □ | □ | □ | □ |

1. **To what extent do you agree with the following statements:**

|  |  | strongly disagree | disagree | slightly disagree | neither agree nor disagree | slightly agree | agree | strongly agree |
| --- | --- | --- | --- | --- | --- | --- | --- | --- |
| ITL1 | I will probably look for a new job in the next year | □ | □ | □ | □ | □ | □ | □ |
| ITL2 | I often think about quitting | □ | □ | □ | □ | □ | □ | □ |

1. **The following questions concern situations where you need help or support in your work.**

If you do not have colleagues tick off ‘not relevant’.

|  |  | Always | Often | Some-times | Seldom | Never/ hardly never | Not relevant |
| --- | --- | --- | --- | --- | --- | --- | --- |
| C33.4 | Is there a good atmosphere between you and your colleagues? | □ | □ | □ | □ | □ | □ |
| C33.6 | Do you feel part of a community at your place of work? | □ | □ | □ | □ | □ | □ |

|  |  | Very satisfied | Satisfied | Unsatisfied | Very unsatisfied |
| --- | --- | --- | --- | --- | --- |
| C34.6 | Regarding your work in general, How pleased are you with your job as a whole, everything taken into consideration? | □ | □ | □ | □ |

|  |  | To a very large extent | To a large extent | Some-what | To a small extent | To a very small extent |
| --- | --- | --- | --- | --- | --- | --- |
| C35.2 | Is it necessary to keep working at a high pace? | □ | □ | □ | □ | □ |
| C35.4 | Does your work require you to take the initiative? | □ | □ | □ | □ | □ |
| C35.5 | Is your work meaningful? | □ | □ | □ | □ | □ |
| CBI16 | Does it drain your energy to work with clients? | □ | □ | □ | □ | □ |
| C35.6 | At your place of work, are you informed well in advance concerning for example important decisions, changes or plans for the future? | □ | □ | □ | □ | □ |
| C35.7 | Does your work have clear objectives? | □ | □ | □ | □ | □ |
| IBenC 2 | Do you get the training you need to do your job well | □ | □ | □ | □ | □ |
| CBI7 | Is your work emotionally exhausting? | □ | □ | □ | □ | □ |
| C35.9 | Are you worried about becoming unemployed? | □ | □ | □ | □ | □ |
| C35.10 | Is your work recognized and appreciated by the management? | □ | □ | □ | □ | □ |
| CBI8 | Do you feel burnt out because of your work? | □ | □ | □ | □ | □ |
| CBI14 | Do you find it hard to work with clients? | □ | □ | □ | □ | □ |
| C35.13 | Do you feel that the work you do is important? | □ | □ | □ | □ | □ |
| C35.14 | Would you recommend a good friend to apply for a position at your workplace? | □ | □ | □ | □ | □ |
| CBI9 | Does your work frustrate you? | □ | □ | □ | □ | □ |
| C35.22 | Do you receive all the information you need in order to do your work well? | □ | □ | □ | □ | □ |
| IBenC 3 | Do you do tasks that are not specifically mentioned in the care plan? | □ | □ | □ | □ | □ |
| C35.25 | Are you treated fairly at your workplace? | □ | □ | □ | □ | □ |
| C35.28 | Do you know exactly what is expected of you at work? | □ | □ | □ | □ | □ |
| CBI15 | Do you find it frustrating to work with clients? | □ | □ | □ | □ | □ |
| C35.30 | Are you worried about it being difficult for you to find another job if you became unemployed? | □ | □ | □ | □ | □ |
| IBenC 4 | Do you do tasks that are not part of your work description but that you can do legally e.g. emptying the letterbox | □ | □ | □ | □ | □ |
| IBenC 5 | Do you do tasks that are not part of your work description and that you are not required to do because you are not qualified for these, e.g. giving an injection to the client as a home health aide | □ | □ | □ | □ | □ |
| C35.31 | Do you have the possibility of learning new things through your work? | □ | □ | □ | □ | □ |
| C35.34 | Is your salary fair in relation to your effort at work? | □ | □ | □ | □ | □ |
| C35.35 | Do you work at a high pace throughout the day? | □ | □ | □ | □ | □ |
| CBI17 | Do you feel that you give more than you get back when you work with clients? | □ | □ | □ | □ | □ |
| C35.37 | Do you feel that your place of work is of great importance to you? | □ | □ | □ | □ | □ |
| C48.4 | To what extent would you say that your immediate superior gives high priority to job satisfaction? | □ | □ | □ | □ | □ |
| C48.5 | To what extent would you say that your immediate superior is good at work planning? | □ | □ | □ | □ | □ |

Information for the IBenC researchers: The following questions are questions only for the managers (will be included in the table above):

|  |  | To a very large extent | To a large extent | Some-what | To a small extent | To a very small extent |
| --- | --- | --- | --- | --- | --- | --- |
| IBenC 6 | In my function as a manager, I’m in touch with the clients | □ | □ | □ | □ | □ |
| IBenC 7 | In my function as a manager, I’m in touch with the family of the clients | □ | □ | □ | □ | □ |
| IBenC 8 | In my function as a manager, I have too much paperwork | □ | □ | □ | □ | □ |

1. **These questions concern the extent you are satisfied with your scheduling scale.** (following items are required only to the care professionals )

In my work situation…

|  |  | Agree | agree somewhat | Disagree somewhat | disagree | Not applicable |
| --- | --- | --- | --- | --- | --- | --- |
| SDS1 | My work schedule is satisfactory | □ | □ | □ | □ | □ |
| SDS2 | I am satisfied with the number of hours I work | □ | □ | □ | □ | □ |
| SDS3 | I am satisfied with the flexibility in overall scheduling | □ | □ | □ | □ | □ |
| SDS4 | I am satisfied with the shifts I work | □ | □ | □ | □ | □ |
| SDS5 | I am satisfied with the flexibility in scheduling weekends/days off | □ | □ | □ | □ | □ |
| SDS6 | I am satisfied with amount of overtime I’m required to work | □ | □ | □ | □ | □ |

# Work and private life

1. **The next two questions are about the way your work affects your private life and family life.**

|  |  | Yes, certainly | Yes, to a certain degree | Yes, but only very little | No, not at all |
| --- | --- | --- | --- | --- | --- |
| C30.1 | Do you feel that your work drains so much of your energy that it has a negative effect on your private life? | □ | □ | □ | □ |
| C30.2 | Do you feel that your work takes so much of your time that it has a negative effect on your private life? | □ | □ | □ | □ |

# The workplace as a whole

1. **The next questions are not about your own job but about the workplace as a whole.**

|  |  | To a very large extent | To a large extent | Some-what | To a small extent | To a very small extent |
| --- | --- | --- | --- | --- | --- | --- |
| C36.1 | Does the management trust the employees to do their work well? | □ | □ | □ | □ | □ |
| C36.4 | Can you trust the information that comes from the management? | □ | □ | □ | □ | □ |
| C36.5 | Are conflicts resolved in a fair way? | □ | □ | □ | □ | □ |
| C36.25 | Is the work distributed fairly? | □ | □ | □ | □ | □ |

# Conflicts and offensive behaviours

|  |  | Yes, daily | Yes, weekly | Yes, monthly | Yes, a few times | No |
| --- | --- | --- | --- | --- | --- | --- |
| C40 | Have you been exposed to undesired sexual attention at your workplace during the last 12 month? | □ | □ | □ | □ | □ |

| If yes, from whom (You may tick off more than one) | □  colleagues | □  Manager/Supervisor | □  subordinates | □  clients/ |
| --- | --- | --- | --- | --- |

|  |  | Yes, daily | Yes, weekly | Yes, monthly | Yes, a few times | No |
| --- | --- | --- | --- | --- | --- | --- |
| C41 | Have you been exposed to threats of violence at your workplace during the last 12 months? | □ | □ | □ | □ | □ |

| If yes, from whom (You may tick off more than one) | □  colleagues | □  Manager/Supervisor | □  subordinates | □  clients/ |
| --- | --- | --- | --- | --- |

|  |  | Yes, daily | Yes, weekly | Yes, monthly | Yes, a few times | No |
| --- | --- | --- | --- | --- | --- | --- |
| C42 | Have you been exposed to physical violence at your workplace during the last 12 months? | □ | □ | □ | □ | □ |

| If yes, from whom (You may tick off more than one) | □  colleagues | □  Manager/Supervisor | □  subordinates | □  clients/ |
| --- | --- | --- | --- | --- |

|  |  | Yes, daily | Yes, weekly | Yes, monthly | Yes, a few times | No |
| --- | --- | --- | --- | --- | --- | --- |
| C43 | Have you been exposed to bullying at your workplace during the last 12 months?  *Bullying means that a person repeatedly is exposed to unpleasant or degrading treatment, and that the person finds it difficult to defend himself or herself against it.* | □ | □ | □ | □ | □ |

| If yes, from whom (You may tick off more than one) | □  colleagues | □  Manager/Supervisor | □  subordinates | □  clients/ |
| --- | --- | --- | --- | --- |

# The care for the clients at home

(Following items are required only for the care professionals)

The statements below concern the care provided to the clients. Please evaluate your care actions in providing care for patients in your latest work shift. Please answer the statements by indicate the alternative that best describes your opinion or experience.

|  |  | Strongly disagree | Disagree to some extent | Neither agree nor disagree | Agree to some extent | Strongly agree |
| --- | --- | --- | --- | --- | --- | --- |
| ICIB01 | I took into account the feelings patients had about their illness/health condition. | □ | □ | □ | □ | □ |
| ICIB02 | I took into account their needs that require care and attention. | □ | □ | □ | □ | □ |
| ICIB03 | Patients assumed responsibility for their care as far as they were able. | □ | □ | □ | □ | □ |
| ICIB04 | I took into account the changes in how they felt. | □ | □ | □ | □ | □ |
| ICIB05 | I took into account their fears and anxieties. | □ | □ | □ | □ | □ |
| ICIB06 | I took into account the way the illness/health condition has affected them. | □ | □ | □ | □ | □ |
| ICIB07 | I took into account the meaning of the illness/health condition to the patient personally. | □ | □ | □ | □ | □ |
| ICIB08 | I took into account their everyday activities (e.g. - leisure activities) outside the care they receive at home. | □ | □ | □ | □ | □ |
| ICIB09 | I took into account their previous experiences of receiving home care. | □ | □ | □ | □ | □ |
| ICIB10 | I took into account patients’ everyday habits during the care (e.g. personal hygiene). | □ | □ | □ | □ | □ |
| ICIB11 | Patients’ families took part in their care if they wanted them to. | □ | □ | □ | □ | □ |
| ICIB12 | I made sure that patients understood the instructions they received. | □ | □ | □ | □ | □ |
| ICIB13 | I gave patients enough information about their illness/health condition. | □ | □ | □ | □ | □ |
| ICIB14 | I took into account patients’ wishes about their care. | □ | □ | □ | □ | □ |
| ICIB15 | Patients took part in decision-making concerning their care. | □ | □ | □ | □ | □ |
| ICIB16 | I took into account the opinions patients expressed about their care. | □ | □ | □ | □ | □ |
| ICIB17 | Patients had the opportunity to make their own decisions on when they receive the care. | □ | □ | □ | □ | □ |
| IBenC 9 | Clients respects me as a part of the health care team | □ | □ | □ | □ | □ |
| IBenC 10 | Clients' family respects me as a part of the health care team | □ | □ | □ | □ | □ |

**Do you have comments about your work or health?**

**Please write here:**

______________________________________________________________________

______________________________________________________________________

______________________________________________________________________

______________________________________________________________________

______________________________________________________________________

______________________________________________________________________

**There are no further questions.**

**Thank you for filling out the questionnaire.**
